# Expression and functional analysis of the Wnt/beta-catenin induced *mir-135a-2* locus in embryonic forebrain development

**DOI:** 10.1186/s13064-016-0065-y

**Published:** 2016-04-05

**Authors:** Giuliana Caronia-Brown, Angela Anderegg, Rajeshwar Awatramani

**Affiliations:** Department of Neurology and Center for Genetic Medicine, Northwestern University Feinberg School of Medicine, 7-113 Lurie Bldg., 303 E. Superior Street, Chicago, IL 60611 USA

**Keywords:** *Wnts*, *Rmst*, *miR-135a*, *Forebrain*, *Lmx1a*, *beta-catenin*

## Abstract

**Background:**

Brain size and patterning are dependent on dosage-sensitive morphogen signaling pathways – yet how these pathways are calibrated remains enigmatic. Recent studies point to a new role for microRNAs in tempering the spatio-temporal range of morphogen functions during development. Here, we investigated the role of *miR-135a*, derived from the *mir-135a-2* locus*,* in embryonic forebrain development.

**Method:**

1. We characterized the expression of *miR-135a,* and its host gene *Rmst*, by in situ hybridization (*ish*). 2. We conditionally ablated, or activated, beta-catenin in the dorsal forebrain to determine if this pathway was necessary and/or sufficient for *Rmst*/*miR-135a* expression. 3. We performed bioinformatics analysis to unveil the most predicted pathways targeted by *miR-135a.* 4. We performed gain and loss of function experiments on *mir-135a-2* and analyzed by *ish* the expression of key markers of cortical hem, choroid plexus, neocortex and hippocampus.

**Results:**

1. *miR-135a*, embedded in the host long non-coding transcript *Rmst*, is robustly expressed, and functional, in the medial wall of the embryonic dorsal forebrain, a Wnt and TGFβ/BMP-rich domain. 2. Canonical Wnt/beta-catenin signaling is critical for the expression of *Rmst* and *miR-135a*, and the cortical hem determinant *Lmx1a*. 3. Bioinformatics analyses reveal that the Wnt and TGFβ/BMP cascades are among the top predicted pathways targeted by *miR-135a*. 4. Analysis of *mir-135a-2* null embryos showed that dorsal forebrain development appeared normal. In contrast, modest *mir-135a-2* overexpression, in the early dorsal forebrain, resulted in a phenotype resembling that of mutants with Wnt and TGFβ/BMP deficits - a smaller cortical hem and hippocampus primordium associated with a shorter neocortex as well as a less convoluted choroid plexus. Interestingly, late overexpression of *mir-135a-2* revealed no change.

**Conclusions:**

All together, our data suggests the existence of a *Wnt/miR-135a* auto-regulatory loop, which could serve to limit the extent, the duration and/or intensity of the Wnt and, possibly, the TGFβ/BMP pathways.

**Electronic supplementary material:**

The online version of this article (doi:10.1186/s13064-016-0065-y) contains supplementary material, which is available to authorized users.

## Background

MicroRNAs are micro-modulators of gene expression, eliciting small changes in the expression of a wide array of targets [1]. In the last ten years, their role in almost every facet of nervous system development and function has been considered including neuronal and glial differentiation, synaptogenesis, and neuro-degeneration [2–10]. Yet only recently, some studies have focused on their role in modulating the dosage or duration of the most fundamental developmental molecules – morphogens [11, 12]. Given the exquisite dosage sensitivity of morphogens, an argument has been proposed that these pathways are prime substrates for microRNA micro-management [12].

Wnt and TGFβ/BMP morphogens expressed by the roof plate, or adjacent neuroepithelium, act through signaling cascades implicated in various facets of dorsal neural tube development [13]. Throughout the Central Nervous System (CNS), various studies have revealed a role for Wnt signaling in the expansion of the brain via increases in cell proliferation and survival [14–18]. Other studies have revealed roles in specification of key neuronal progenitor types, as well as in the timing of neurogenesis [16, 19–22]. Additionally, several studies have suggested that the dosage of the Wnt pathway is critical for normal specification, neurogenesis and differentiation [15, 19, 23, 24]. TGFβ/BMP signaling has also been implicated in proliferation, specification, neurogenesis and gliogenesis [25–31]. Akin to the Wnt pathway, several studies have suggested that the TGFβ/BMP pathway is exquisitely dosage sensitive [32, 33]. Despite an emerging literature on the cross talk between these two key pathways [31, 34], the potential nodes of intersection and their net molecular outputs remain to be fully elucidated. It is likely that the molecular synchronization of these pathways is required for dorsal neural tube development.

The cortical hem, positioned adjacent to the hippocampus, between the choroid plexus on the medial side and the cortical neuroepithelium on the lateral side, is a Wnts and TGFβ/BMPs-rich embryonic structure [29, 35, 36]. The hem has been demonstrated to specify the hippocampus primordium [37–42], to serve as a source of Cajal-Retzius cells [43], to be required for choroid plexus formation [16, 29, 36] and to play a role in regulating the size and patterning of the neocortex [44].

Previously, we identified a microRNA, *miR-135a,* whose expression was correlated to the long non-coding transcript *Rhabdomyosarcoma 2 associated transcript* (*Rmst)*. We deduced that *miR-135a* was derived from *mir-135a-2* locus embedded in *Rmst* and we demonstrated that *Rmst* and *miR-135a* are co-expressed in the ventral midbrain, isthmus, as well as dorsal regions of the neural tube [11]. At least in the midbrain, modest and early overexpression of this microRNA yields phenotypes consistent with a reduction of Wnt signaling [11]. Given the potential importance of this microRNA, we have explored its expression, activity and induction in the dorsal forebrain as well as generated *mir-135a-2* knockout and overexpressor mice. We reveal that *miR-135a* is strongly expressed, and is functional, in the medial wall of the telencephalon including the cortical hem and hippocampus primordium, but more weakly expressed in the choroid plexus and in the neocortex. We show that canonical Wnt/beta-catenin signaling is critical for *Rmst* and *miR-135a* expression, and also for *Lmx1a* expression, a key cortical hem determinant. While *mir-135a-2* loss of function did not result in appreciable changes in the cortical hem and neocortex sizes or in choroid plexus complexity, its modest over-expression resulted in smaller cortical hem and neocortical domains and also in a less convoluted choroid plexus. All together, our data lead us to conclude that this Wnt induced microRNA is a potential modulator of the Wnt and TGFβ/BMP signaling pathways during dorsal forebrain development.

## Methods

### Nomenclature

miRbase uses a 3 or 4 letter prefix to designate the microRNA species, such that ‘mmu’ refers to the mouse. The un-capitalized ‘mir’ refers to the pre-microRNA (*mmu-mir-135*). In this manuscript, we have only investigated the murine *mir-135* therefore we have omitted the prefix. Distinct genomic loci that belong to the same family (*mir-135*) are typically indicated with an additional letter and number such as *mir-135a-1*, *mir-135a-2* and *mir-135b*. The capitalized ‘miR’ refers to the mature form (*miR-135*). *mir-135a-1* and *mir-135a-2* give rise to only one mature form called *mmu-miR-135a-5p*. For simplicity, we will refer to the mature form as *miR-135a.* However, our experiments on *mir-135a-2* knockout mice imply that the predominant mature form of *miR-135a* in the dorsal forebrain is produced from the *mir-135a-2* locus.

### Mouse lines

Animals were maintained in compliance with National Institutes of Health guidelines. The Northwestern University IACUC approved the protocols for this study. E0.5 designates the morning of the day when a vaginal plug was detected. For beta-catenin gain and loss of function experiments, *Ctnnb1*^*lox(ex3)*^ [45] or beta-catenin floxed mice (*Ctnnb1*^*flox/flox*^) [46], were crossed with *Emx1::IRES-Cre* [47] and embryos were used for in situ hybridization (*ish*) or RT-qPCR experiments. The *miR-135a* “sensor” construct was previously described [11]. To evaluate *miR-135b* expression, we used *mir-135b*^*flox/flox*^ mice (Jackson lab) [48], which harbor a LacZ cassette and crossed them to wild type females. E12.5 embryos of the *mir-135b*^flox/+^ genotype were stained for Xgal as previously described [49]. To generate the *mir-135a-2* knockout mice, we utilized ZFN technology (Sigma). One advantage of this approach is that no selection cassette or residual FRT, or loxP sites, will remain in the intron, and the resultant deletion will be clean. Sixteen different ZFNs were custom designed to bind and cleave the *mir-135a-2* locus, within 100 bp upstream or downstream of the stem-loop precursor. The ZFN (GCCATCAGGATAGCnAACTATAGCCTGTGGAC) that demonstrated the highest activity, in an in vitro Mouse *Neuro2a* cell screen, was chosen for large-scale production and microinjection in mice. The ZFN mRNA was diluted to 2.5 ng/μl in injection buffer and microinjected into early stage FVB embryos. 152 mice were screened with ZFN-F: GGTCCTCGTAGCGAAGAATG and ZFN-R: AATCGGTGGTCAGGAAGATG PCR primers. Five heterozygous mice were identified with one wild type allele and one allele containing a deletion near the *mir-135a-2* locus. After sequence analysis, we found that each deletion was unique and ranged from 2 bp to 294 bp. Line #4 had the largest deletion, which removed the entire *mir-135a-2* precursor, and was used for the experiments here described. RT-qPCR with TaqMan primers was used to confirm drastic reduction of the mature form *miR-135a.*

Sensor transgenic embryos (*n* = 4), and *mir-135a-2* knockout (*mir-135a-2KO*) mice were generated at the Northwestern Transgenic and Targeted Mutagenesis Laboratory.

Generation and genotyping of *mir-135a-2* overexpressor transgenic mouse line have been previously described in [11]. To generate conditional *mir-135a-2OE* embryos, *mir-135a-2OE* mouse line was crossed with *Emx1::IRES-Cre* (henceforth *Emx1::Cre*) [47] or *Nestin::Cre* (henceforth *Nes::Cre*) [50]. As controls for these experiments, we used littermates negative for *Cre*. To generate *Emx1::Cre*;*mir-135a-2OE*;*mir-135a-2*^+/-^ embryos, *Emx1::Cre*;*mir-135a-2OE* adult mice were crossed with *mir-135a-2* knockout mice.

### Tissue processing

Brains were fixed with 4 % PFA and either embedded in 30 % sucrose-10 % gelatin-PBS and sectioned with a Leica SM2010R sliding microtome, or in OCT and sectioned with a Leica cryostat. Sections (20–40 μm) were processed for *ish* with Digoxigenin (Dig)-labeled riboprobes [43]. Bound Dig was detected with anti-Dig antibody (1:5000, Roche). To detect *Rmst*, we used two probes as described in [11]. To detect *miR-135a*, we performed locked-nucleic acid (LNA) *ish* with Exigon probe # 39037-01 and followed recommended protocol for non-radioactive hybridization by Dr. Wigard Kloosterman, the Plasterk Group, Hubrecht Laboratory, Utrecht, The Netherlands, with the following modifications: no de-paraffinization step; PK treatment 5’–10’ at 37C (20-40 μm sections); T hyb 53C; probe [25nM]; blocking solution 10 % lamb serum-TBST; anti-Dig-AP was diluted 1:5000 in 1 % lamb serum-TBST. eGFP expression, in double transgenic embryos, was detected by immunofluorescence with anti-GFP rabbit polyclonal (1:1500, Invitrogen) without antigen retrieval on 20 μm cryostat sections. Secondary antibody was donkey anti-rabbit 488 (Invitrogen). No immunostaining was necessary to detect tdTomato expression. For Xgal staining, brains were lightly fixed in 2 % PFA-PIPES solution, washed in PBS and Xgal stained from few hours to overnight at 37C. To generate coronal sections, after Xgal staining, brains were fixed in 4 % PFA overnight and processed for cryostat sectioning (50 μm). For immunohistochemistry (IHC) assay, brains were also fixed in 4 % PFA overnight and sectioned at 20 μm with a Leica cryostat. After citrate antigen retrieval, sections were incubated with anti-phospho-Smad 1/5/8 rabbit polyclonal (1:50, Cell signaling). Secondary antibody was biotinylated anti-rabbit polyclonal from ABC KIT (1:200, Vectastain). In this study, gene expression comparisons between control and mutant mice were based on at least 3 brains per group per age for each gene.

### Quantification of cortical tissue and cortical hem area

At E12.5, the length of the neocortex was measured from the pallium-subpallium boundary (PSB), chosen as a landmark, to the cortical hem. Quantification was performed at three levels of the brain, 80 μm apart, along the rostro-caudal axis. At the same stage, we additionally quantified the cortical hem area (*Lmx1a*+). For all of the measurements, we used ImageJ software (series 1.4, NIH, public domain). Data are expressed as a mean ± the standard error (SEM) (*n* = 3).

### RT-qPCR

For RT-qPCR experiments, dorsal or ventral forebrain tissue was dissected from controls (wild types) and mutants (*Emx1::Cre*;*Ctnnb1*^*lox(ex3)*^*, Emx1::Cre*;*Ctnnb1* cKO, *mir-135a-2* knockout and overexpressor) (*n* = 3). Briefly, E12.5 embryos were collected in ice cold PBS, the forebrain was exposed, and a piece of the dorsal, or ventral, forebrain tissue was snipped with forceps and processed for RNA extraction. Total RNA, including small RNAs, was extracted using the *mir*Vana kit (Ambion). To quantify *miR-135a* and *miR-135b* expression levels, we used the TaqMan PCR Assay (ID 000460 and ID 002261, Applied Biosystems) and normalized our data to microRNA *sno202* (ID 001232, Applied Biosystems).

### Statistical analysis

To determine statistical significance of our quantification experiments, we first determined if data followed the normal distribution by the Anderson-Darling Test for Normality. All of our data sets had a p value > 0.05, indicating normality. To assess the statistical significance of changes in the cortical hem area and neocortical domain length, the two experimental groups (control and mutant mice) were compared with two samples equal variance, two tailed, Student’s *t*-test. To calculate the relative fold changes in *miR-135a* and *miR-135b* expression by RT-qPCR experiments, we applied the comparative C(T) method also referred to as the 2 (-DeltaDeltaC(T)) method [51] and normalized our data to microRNA *sno202*. Unpaired Student’s *t* test was applied to determine statistical significance.

### Bioinformatics analysis

To determine *miR-135a* most predicted targeted pathways, we used the Diana-miRPath, a microRNA pathway analysis web server that combines predicted and validated microRNA targets in CDS or 3’-UTR regions with sophisticated merging and meta-analysis algorithms [52].

## Results

### Rmst and miR-135a expression and activity in the embryonic forebrain

**Fig. 1:**
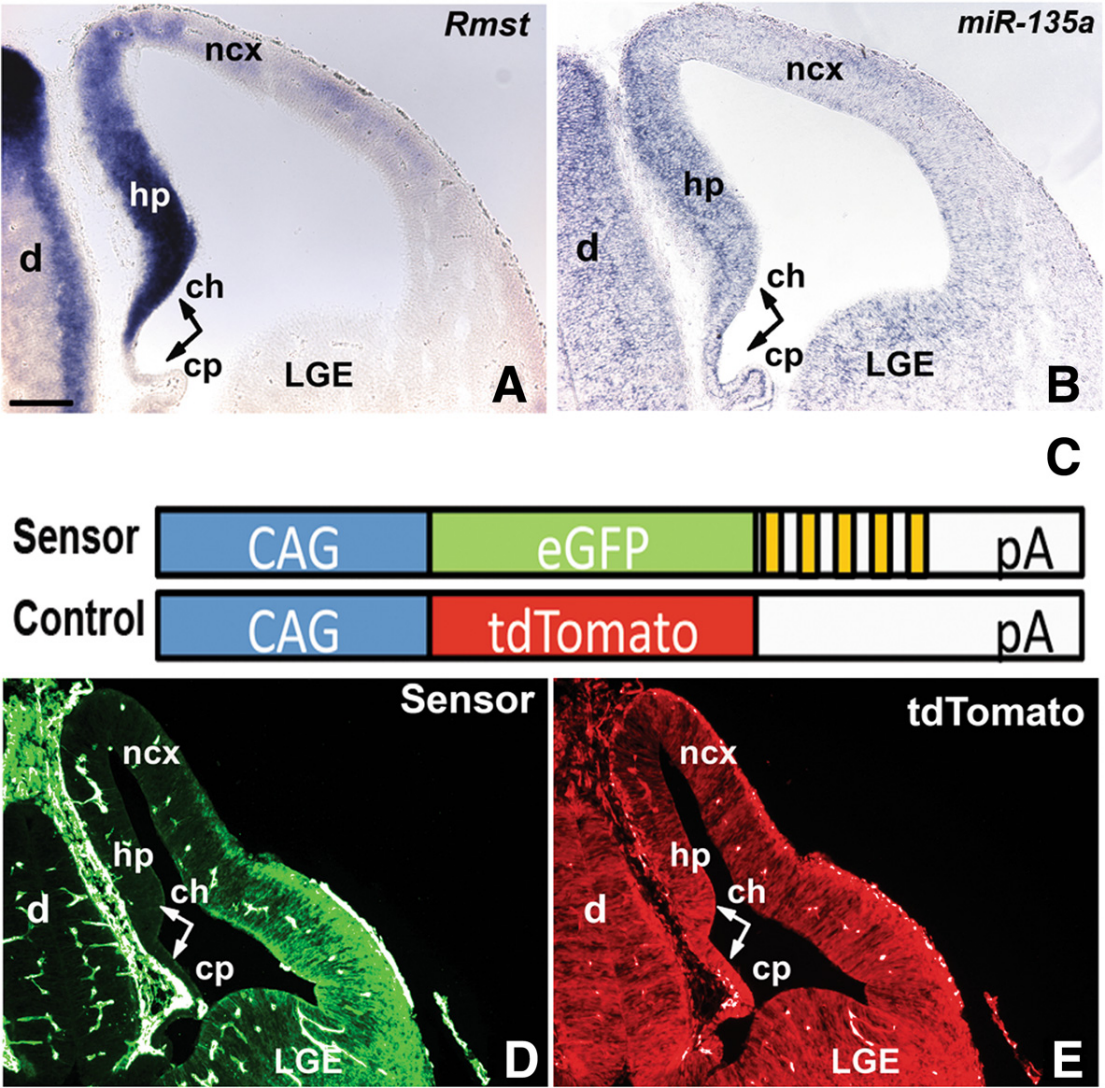
*Rmst and miR-135a* expression and activity in the embryonic dorsal forebrain. (**a**-**b** and **d**-**e**). Forebrain coronal sections of E12.5 wild type (**a**, **b**) and double transgenic mice (**d**, **e**). **a** *ish* for *Rmst*. **b** LNA *ish* for mature *miR-135a.* **d**, **e** Double transgenic embryos harboring eGFP “sensor” and tdTomato control constructs represented in **c** (cartoon; yellow bars indicate *miR-135a* binding sites). **d** eGFP immunolabeling **e** tdTomato fluorescence. ch, cortical hem; hp, hippocampus primordium; cp, choroid plexus; LGE, lateral ganglionic eminences; ncx, neocortex; d, diencephalon. Scale bar 100 μm

Previously, we identified *miR-135a* through a screen for microRNAs that were robustly expressed in the Wnt-rich ventral midbrain region of the embryonic Central Nervous System (CNS) [11]. We provided evidence that *miR-135a* expression was correlated with *Rmst*, and deduced that *miR-135a* was derived from *mir-135a-2* locus located in the final detected intron of *Rmst* [11, 53]. Since the embryonic dorsal forebrain is known to be a Wnt-rich region and dependent on Wnt signaling [36, 54, 55], we determined if *Rmst* and *miR-135a* were also co-expressed in this region of the CNS. We therefore first characterized *Rmst* expression at embryonic and adult stages (Fig. 1 and Additional file 1: Figure S1). In embryonic forebrain sections *Rmst* is robustly detected in the medial wall of the telencephalon, encompassing a Wnt and TGFβ/BMP-rich signaling center, the cortical hem [35, 56]. At E12.5, *Rmst* was strongly expressed in the dorsal telencephalon in a medial^High^ to dorso-lateral^Low^ gradient (Fig. 1a). Expression was robust in the cortical hem and the adjacent hippocampus primordium. A very weak hybridization signal was detected in the choroid plexus and along the neocortical domain. *Rmst* was also strongly expressed in the septum, diencephalon, and eminentia thalami. At later stages, *Rmst* expression was localized to various forebrain nuclei, and the fimbria (Additional file 1: Figure S1). We next performed Locked Nucleic Acid (LNA) *ish* experiments to detect mature *miR-135a*, focusing on the embryonic forebrain. We found that this microRNA species, like *Rmst*, was expressed in the medial wall of the telencephalon in a medial^High^ to dorso-lateral^Low^ gradient (Fig. 1b). Expression was robust in the cortical hem and the adjacent hippocampus primordium. A very weak hybridization signal was detected in the choroid plexus and along the neocortical domain. Some signal was also detected in the LGE, however, since *Rmst* is not expressed in this region, this signal could represent cross hybridization with a closely related microRNA. Alternatively, in this region *miR-135a* and *Rmst* expression could be uncoupled.

To demonstrate that *miR-135a* is functional in these domains, we generated double transgenic embryos harboring a “sensor” and a control transgene. A “sensor” construct contains a constitutively expressed reporter gene (eGFP), under control of a CAG promoter, and multiple binding sites for *miR-135a* in the 3’ UTR region (Fig. 1c, cartoon, yellow bars). In cells expressing *miR-135a*, its perfect complementarity to sequences in the 3’ UTR should result in suppression of the eGFP reporter. A control transgene construct contains a tdTomato reporter, but lacks the *miR-135a* binding sites and, therefore, should be constitutively active. In E12.5 double transgenic embryos, we found that the eGFP reporter (Fig. 1d), but not tdTomato (Fig. 1e), was selectively reduced in the medial wall of the telencephalon where *miR-135a* is strongly expressed, as well as in the choroid plexus and in the dorsal neocortical domain, where *Rmst* and *miR-135a* are more weakly expressed. eGFP was also not detected in the diencephalon (Fig. 1d). Thus, *miR-135a* is expressed, and displays activity, in the embryonic dorsal forebrain.

### *beta-catenin signaling induces Rmst* and *miR-135a expression*

**Fig. 2:**
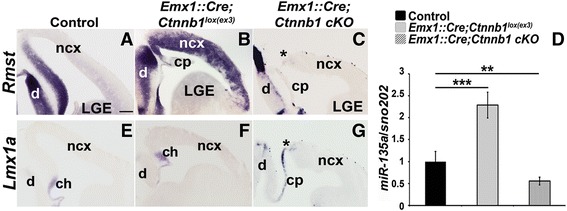
*Rmst, miR-135a* and *Lmx1a* are altered in beta-catenin mutants. **a**-**c** *Rmst* expression in E12.5 wild type control (**a**), *Emx1::Cre*;*Ctnnb1* ^*lox(ex3)*^ (**b**) and *Emx1::Cre*;*Ctnnb1* cKO mutant (**c**) brains. **d** Quantification of *miR-135a* expression levels by RT-qPCR on E12.5 dissected dorsal telencephalon of control (wild type), *Emx1::Cre*;*Ctnnb1* ^*lox(ex3)*^ and *Emx1::Cre*;*Ctnnb1* cKO mutants (*n* = 3). *miR-135a* fold induction was normalized to microRNA *sno202*. **e**-**g** *Lmx1a* expression in E12.5 controls (**e**) and beta-catenin mutant (**f** and **g**) brains. Asterisks in **c** and **g** highlight remaining cortical hem tissue in *Emx1::Cre*;*Ctnnb1*cKO mutants. ***, p value <0.001; **, *p* < 0.01; *, *p* < 0.05 cp, choroid plexus; ncx, neocortex; LGE, lateral ganglionic eminences; d, diencephalon. Scale bar 100 μm

Previously, we reported that in the embryonic midbrain, the transcription factor *Lmx1b* induces Wnt1/Wnt signaling as well as *Rmst* and *mir-135a-2* locus [11]. Since *Lmx1b* and *Lmx1a* are partially redundant, we postulated that *Lmx1a* might also be a regulator of *Rmst* and *mir-135a-2*. However, since, in the dorsal forebrain, *Rmst* and *miR-135a* expressions exceed the *Lmx1a* domain, one possibility is that in addition to *Lmx* genes, Wnt signaling abets the induction of the *Rmst* and *mir-135a-2* locus. To address this question, we performed gain and loss of function experiments of beta-catenin, a key effector of Wnt signaling pathway [57]. *Emx1::Cre* mouse line was used to drive recombination throughout the dorsal, but not ventral, forebrain [47]. For gain of function experiments, by conditionally deleting beta-catenin exon 3 (*Ctnnb1*^*lox(ex3)*^), which encompasses GSK3β phosphorylation sites, we effectively elevated Wnt signaling [23, 45]. In mutants (*Emx1::Cre*;*Ctnnb1*^*lox(ex3)*^) (Fig. 2b), but not in controls (Fig. 2a), *Rmst* was detected throughout the dorsal forebrain*.* Conversely, for loss of function experiments, we conditionally removed exons 2-6 of beta-catenin to prevent formation of a functional beta-catenin protein thus impairing Wnt signaling [46]. In mutants (*Emx1::Cre*;*Ctnnb1* cKO) (Fig. 2c), but not in controls (Fig. 2a), we observed drastic loss of *Rmst* expression throughout the dorsal forebrain and in the remaining cortical hem tissue (Fig. 2c, asterisk). *miR-135a* expression levels, quantified by RT-qPCR on E12.5 dissected dorsal telencephalon, were strongly induced in *Emx1::Cre*;*Ctnnb1*^*lox(ex3)*^ mutants, and reduced in *Emx1::Cre*;*Ctnnb1* cKOs (Fig. 2d). Overall, these data demonstrate that beta-catenin signaling is necessary and sufficient for *Rmst* and *miR-135a* expression in the dorsal forebrain.

### beta-catenin signaling is necessary for Lmx1a expression

We also determined if *Lmx1a*, a key cortical hem determinant [58], is a target of Wnt/beta-catenin signaling in the forebrain, as in other brain regions [11, 22, 23]. While in *Emx1::Cre*;*Ctnnb1*^*lox(ex3)*^ embryos (Fig. 2f), we did not observe a drastic change in the cortical hem size (*Lmx1a+*, Fig. 2f and *Wnt3a+*, Additional file 2: Figure S2) or in *Lmx1a* expression (Fig. 2f) with respect to controls (Fig. 2e), in *Emx1::Cre*;*Ctnnb1* cKOs, we observed a drastic reduction of *Lmx1a* signal in the remaining cortical hem tissue (Fig. 2g, asterisk). *Lmx1a* was detected in the choroid plexus of both mutants (Fig. 2f and g). These results suggest that Wnt/beta-catenin signaling is necessary, but not sufficient, for *Lmx1a* expression in the dorsal forebrain. Given that *Lmx1a* in part functions to repress *Lhx2* [58], a negative regulator of the hem [39], Wnt/beta-catenin induction of *Lmx1a* is likely to be an important event in the cortical hem establishment and/or maintenance.

### miR-135a is predicted to target the Wnt and TGFβ/BMP pathways, but loss of function does not affect dorsal forebrain development

To begin to elucidate *miR-135a* functions, we performed bioinformatics analysis to determine the most common pathways targeted by *miR-135a*. To do that, we took advantage of the Diana–miRPath software [52], which utilizes predicted, and validated, microRNA targets to perform a hierarchical clustering of microRNA and pathways based on their interactions. We found that Wnt and TGFβ/BMP signaling pathways are among the top pathways targeted by *miR-135a* with an extremely high statistical significance (p value of approximately 2.9E-07 and 3.6E-10, respectively) (Additional file 3: Figure S3A). It is worth noticing that the genes targeted by *miR-135a* in the TGFβ/BMP (Additional file 3: Figure S3B) and Wnt pathways (Additional file 4: Figure S4) include ligands, receptors and downstream transcriptional regulators suggesting that this microRNA likely acts through multiple levels of the Wnt and TGFβ/BMP cascades to modulate the outcome of their signaling.

**Fig. 3:**
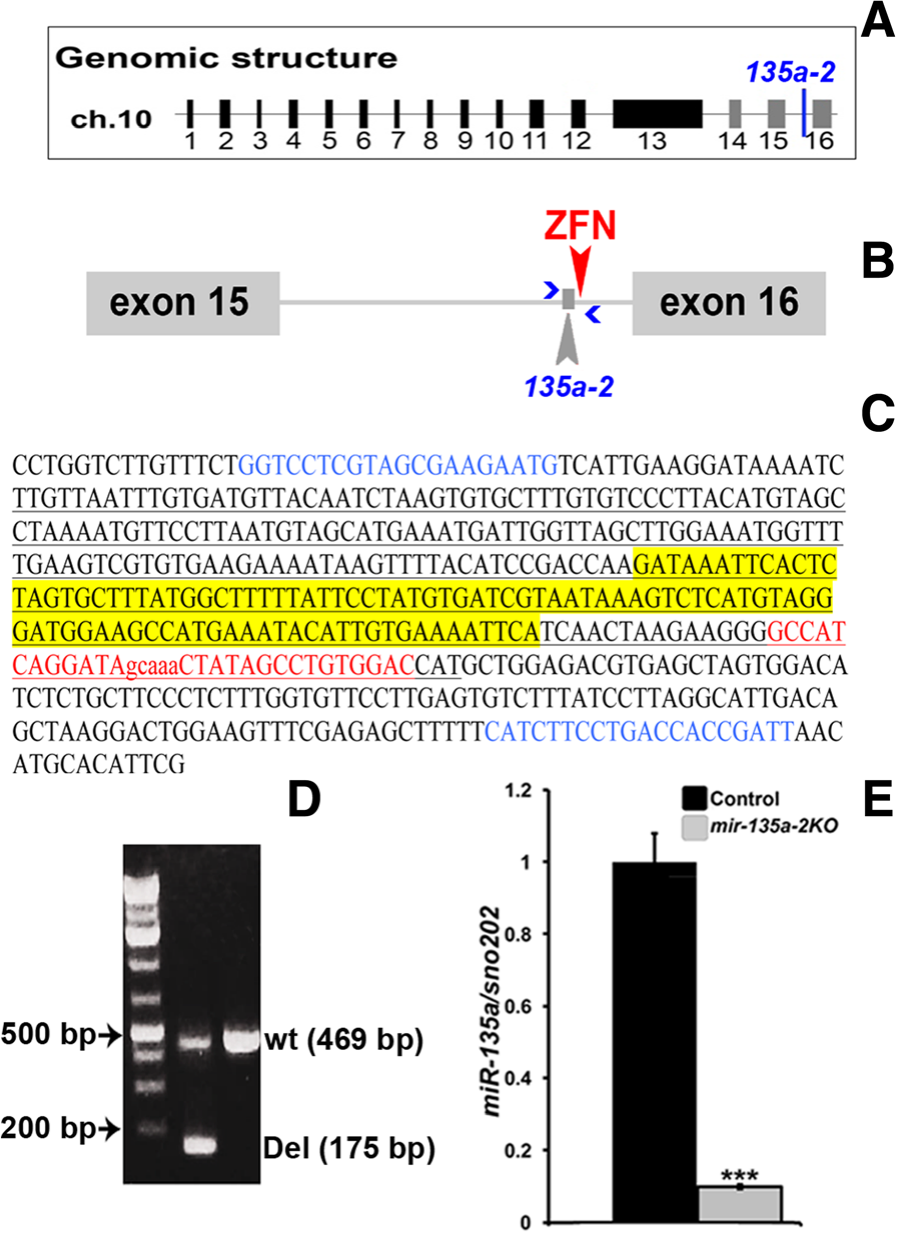
*mir-135a-2* deletion by ZFN nuclease. **a** Representation of *Rmst* genomic structure. *mir-135a-2* is indicated between exons 15 and 16. **b** Cartoon of the last intron of *Rmst* showing the location of *mir-135a-2* and the ZFN cut site. Blue arrows show primers designed to detect the wild type and deleted bands. **c** Sequence of *Rmst* genomic region in which *mir-135a-2* is embedded. Deleted region is underlined; PCR primers are indicated in blue; *mir-135a-2* precursor sequence is in yellow; the ZFN binding site is in uppercases red and in lowercases is the ZFN cut site. **d** PCR showing the wild type and the ZFN deleted bands. **e** RT-qPCR showing a drastic reduction of *miR-135a* in E12.5 dissected dorsal forebrain tissue from control and *mir-135a-2* null mice (*n* = 3). Data are shown as a fold change and have been normalized to microRNA *sno202*. ***, *p* value <0.001

To determine the role of *miR-135a* in embryonic dorsal forebrain development, we generated a mouse line in which *mir-135a-2* was deleted by pronuclear injection of a specific Zn finger nuclease (Fig. 3a and b), designed to cleave <100 bp from the mature sequence. Of ~60 pups examined, 1 harbored a ~294 bp deletion (Fig. 3c and d) and was used to generate a line for the experiments here described. RT-qPCR on dissected E12.5 dorsal forebrain tissue confirmed a drastic reduction in *miR-135a* expression levels (Fig. 3e). We thus deduced that, in the dorsal forebrain akin to the ventral midbrain, *miR-135a* is predominantly produced from *mir-135a-2* locus.

**Fig. 4:**
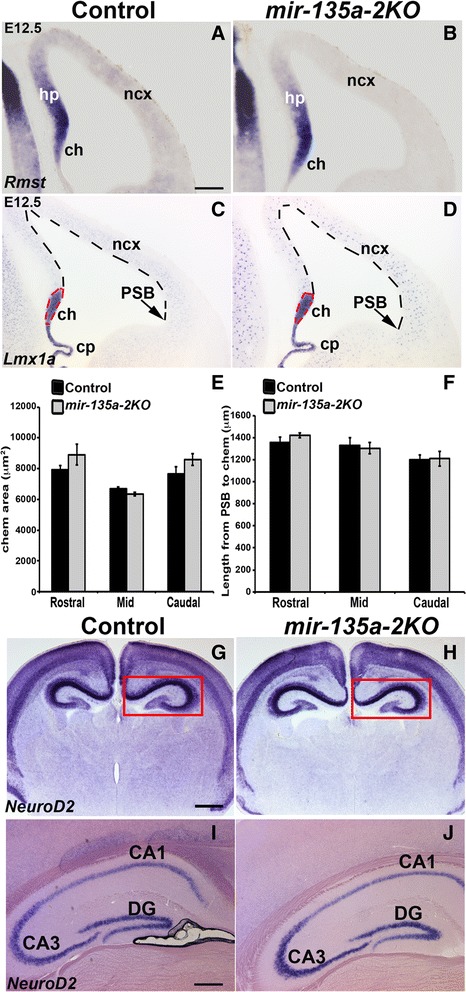
*mir-135a-2* loss of function characterization. **a**-**b** E12.5 coronal sections of control (*mir-135a-2* ^*+/-*^) and *mir-135a-2*KO brains (*mir-135a-2* ^*-/-*^) processed for *ish* for *Rmst* showing no change in its expression. **c**-**d** *ish* for *Lmx1a* in control (**c**) and *mir-135a-2*KO (**d**) brains. Dashed lines highlight the cortical hem area and the neocortical domain size. **e** Quantification of the cortical hem area (μm^2^ ± SEM) along the rostro-caudal axis (*n* = 3). **f** Quantification of the neocortex length (μm ± SEM) from the PSB to the hem at the same levels (*n* = 3). **g**-**j** *ish* for *NeuroD2* on coronal sections of post-natal P1 (**g**-**h**) and adult (**i**-**j**) control and mutant brains showing no change in hippocampus morphology (boxed in **g** and **h**). ch, cortical hem; cp, choroid plexus; hp, hippocampus; PSB, pallium-subpallium boundary; ncx, neocortex; CA1, CA3, hippocampus fields; DG, dentate gyrus. Scale bar 100 μm in panels **a**-**d**, 400 μm in panels **g** and **h**; 200 μm in panels **i** and **j**

Because a growing literature has described interactions between microRNAs and long non-coding RNAs (lncRNAs) as a novel mechanism to regulate gene expression as well as microRNA function [59–61], we investigated the possibility of an interaction between *Rmst* and *miR-135a*. A search on miRcode web site, which represents a comprehensive map of putative microRNA target sites across the GENCODE long non-coding transcriptome [62], indicated lack of *miR-135a* responsive elements in *Rmst*. To confirm the bioinformatics prediction, we performed *ish* for *Rmst* on E12.5 coronal sections of *mir-135a-2* knockout mice and we did not observe any change in its expression (Fig. 4a and b). These data seem to therefore exclude the possibility of *Rmst* being a potential target of *miR-135a.* Next, we performed in situ hybridization with *Lmx1a* (Fig. 4c and d), a marker for the cortical hem and the choroid plexus to investigate any possible change in proper development of these Wnt and TGFβ/BMP responsive domains. We did not, however, observe any apparent change in the cortical hem size (Fig. 4d) as was also confirmed by quantification analysis at three levels of the forebrain, equally spaced along the rostro-caudal axis (referred to as Rostral, Mid and Caudal) (Fig. 4e). No difference was detected in the extent of the cortical domain (Fig. 4d), which was again measured at three levels of the forebrain, equally spaced along the rostro-caudal axis (Rostral, Mid and Caudal) from the inflection that marks the pallium-subpallium boundary (PSB), chosen as a landmark, to the cortical hem (Fig. 4f). No change in the choroid plexus complexity was observed (Fig. 4d).

The cortical hem is a signaling center known to induce and pattern the adjacent hippocampus [38, 41]. We therefore performed in situ hybridization with neuronal marker *NeuroD2* to assess any morphological changes in the hippocampal complex at post-natal and adult stages of *mir-135a-2* knockouts. No changes were observed (Fig. 4g-j). These findings clearly demonstrate that, at least by these criteria, *mir-135a-2* loss of function does not alter forebrain development.

**Fig. 5:**
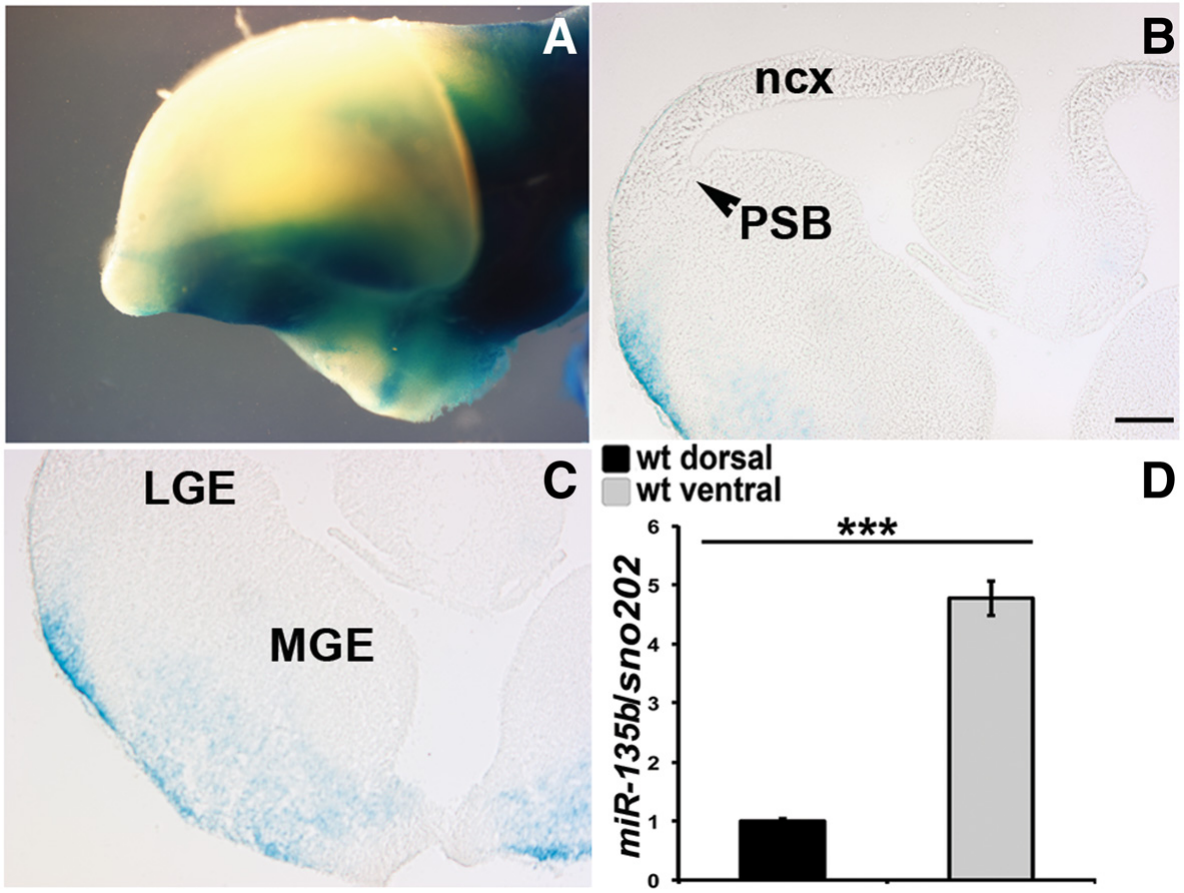
*miR-135b* expression in the embryonic forebrain. **a**-**c** Xgal staining of E12.5 whole mount (lateral view) (**a**) and E12.5 coronal sections (**b**-**c**), showing expression of *lacZ* exclusively in the ventral forebrain of *mir-135b* ^flox/+^ mice harboring a LacZ reporter gene in the *mir-135b* locus. **d** RT-qPCR on wild type dorsal and ventral forebrain tissue samples (*n* = 3). *miR-135b* fold induction was normalized to microRNA *sno202.* ***, *p* value <0.001. LGE, lateral ganglionic eminences; MGE, medial ganglionic eminences; PSB, pallium-subpallium boundary; ncx, neocortex. Scale 200 μm in panels **b** and **c**

microRNAs often display redundancy with family members [63, 64]. To determine whether *miR-135b*, a closely related microRNA was expressed in a similar domain, we obtained mice in which LacZ had been inserted into the *mir-135b* locus. In E12.5 whole mount (Fig. 5a) and forebrain sections (Fig. 5b and c), we did not observe any LacZ staining in the dorsal forebrain (Fig. 5a and b) whereas we found LacZ expression in cells emanating from the ganglionic eminences in the ventral forebrain (Fig. 5c). Consistent with this, RT-qPCR experiments for *miR-135b* on wild type dorsal and ventral forebrain dissected tissues, revealed a ventral enrichment of this microRNA (Fig. 5d). These data suggest that *miR-135b* does not serve a redundant function in the dorsal forebrain.

### Early mir-135a-2 overexpression affects dorsal forebrain development

**Fig. 6:**
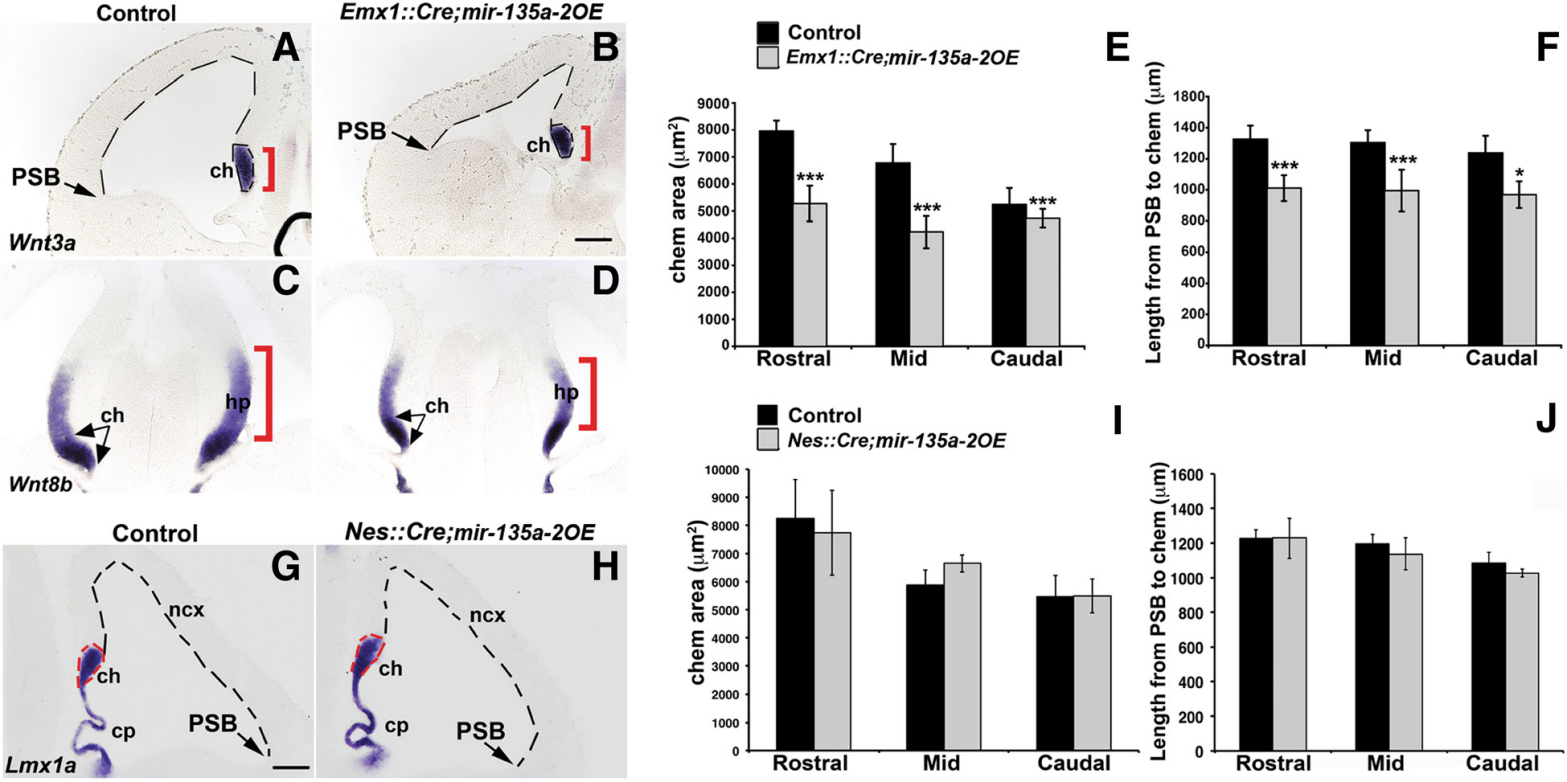
Early, but not late, *mir-135a-2OE* resembles mutants with Wnt deficits. **a**-**d** Coronal sections of E12.5 control (*mir-135a-2OE*) and mutant (*Emx1::Cre*;*mir-135a-2OE*) brains. *Wnt3a* (**a**, **b**) and *Wnt8b* (**c**, **d**) mark the cortical hem (*Wnt3a +* and *Wnt8b+,* brackets and arrows) and the hippocampus (hp) primordium (*Wnt8b+,* brackets). Dashed lines highlight the neocortex length from the PSB to the hem. **g**-**h** *Lmx1a* expression in E12.5 control (*mir-135a-2OE*) and *Nes::Cre*;*mir-135a-2OE* mutant brains. Dashed lines highlight the cortical hem area and the neocortical domain size. **e**-**f** and **i**-**j** Quantifications of the cortical hem area (μm^2^ ± SEM) along the rostro-caudal axis (*n* = 3) and the neocortex length (μm ± SEM) from the PSB to the hem at the same levels (*n* = 3) in *Emx1::Cre*;*mir-135a-2OE* (**e** and **f**) and *Nes::Cre*;*mir-135a-2OE* (**i** and **j**) mutants. ***, *p* <0.001; *, *p* < 0.05. ch, cortical hem; PSB, pallium-subpallium boundary; ncx, neocortex. Scale bar 100 μm in panels **a**, **b** and **g**, **h**; 50 μm in panels **c** and **d**

To complement our loss of function results, we took advantage of previously generated transgenic mice to conditionally express *mir-135a-2* for gain of function experiments. This time, we reasoned that if the *Wnt/miR-135a* circuitry identified in the midbrain [11] is functionally conserved in the forebrain, *mir-135a-2* overexpression might result in Wnts related phenotypes. To test this hypothesis, mice in which *mir-135a-2* precursor expression is under control of a CAG promoter [11] (henceforth *mir-135a-2OE*), were crossed with *Emx1::Cre* line [47] to overexpress *mir-135a-2* throughout the *Emx1* domain of the dorsal, but not ventral, forebrain as early as E9.5 [43]. RT-qPCR on dissected E12.5 dorsal forebrain tissue confirmed that *miR-135a* expression levels, in *Emx1::Cre*;*mir-135a-2OE* mutants, were 1.5 fold more than in controls (Additional file 5: Figure S5). In such mutants we observed a clear reduction in the size of the cortical hem (*Wnt3a+*, *Wnt8b+*) and the hippocampus primordium (*Wnt8b+*) (Fig. 6a-d). Additionally, we observed a reduction in size of the neocortical domain (Fig. 6a-b, dashed lines), a phenotype previously shown in mice with genetic ablation of the cortical hem [44, 65], but not reported in *BmpRIA/IB* cKOs which display a smaller cortical hem [42]. The choroid plexus, normally specified by TGFβ/BMPs [29, 42] and demarcated by *rTtr1*, was overall less convoluted (Additional file 6: Figure S6). Altogether, *Emx1::Cre*;*mir-135a-2OE* mutants show characteristics previously described in mice deficient for Wnt or TGFβ/BMP activity, or in which the cortical hem has been genetically ablated [16, 29, 42, 44, 65, 66], although they display a milder phenotype.

Next, we quantified the cortical hem area along the rostro-caudal axis. We estimated a reduction in its size of ~34 % at rostral level, ~38 % at mid level and ~18 % at caudal level (Fig. 6e) in mutants compared to controls. Finally, we quantified the extent of the neocortical domain and found a significant reduction in size of the mutant neocortices in comparison to controls (Fig. 6f). Since the cortical hem and the hippocampus primordium are reduced in *Emx1::*Cre;*mir-135a-2OE* embryos, these mutants showed a significant reduction of all hippocampal structures (CA fields and dentate gyrus) from post-natal stage P1 (Additional file 7: Figure S7, A-B and E) to adulthood (Additional file 7: Figure S7C and D) when compared to controls.

Finally, we examined the expression of several bioinformatically predicted *miR-135a* targets. Of these, only phospho-Smad (1/5/8) and *Msx2* revealed consistent changes, showing apparent reduction in their level and expression domain extent (Additional file 8: Figure S8). Their reduction might be due to direct repression, overall net down regulation of these pathways, or both.

### Late mir-135a-2 overexpression does not affect dorsal forebrain development

Conditional transgenes in neural progenitor cells have been associated with non-specific phenotypes [67]. To rule out non-specific effects of *mir-135a-2* overexpression, we also overexpressed *mir-135a-2* by using *Nes::Cre* driver [50], which like *Emx1::Cre,* is active in the hippocampus primordium, along the neocortical domain, and reported to mediate Cre recombination in the hem at ~ E12.5 [10, 68–71]. *Nes::Cre* driven overexpression of *mir-135a-2* was additionally useful to determine whether the phenotype resulting from *mir-135a-2* overexpression in *Emx1::Cre*;*mir-135a-2OE* mice, was time sensitive. In E12.5 (Fig. 6g and h) and E13.5 (Additional file 9: Figure S9) *Nes::Cre*;*mir-135a-2OE* mutants, we did not observed microcephaly. Both the cortical hem and neocortical sizes were not affected (Fig. 6i and j). Also, no change was observed in the choroid plexus (Fig. 6g and h). These data suggest that late overexpression of this microRNA in neural progenitors, does not affect forebrain development, and that the transgene does not appear to display significant toxicity in neuronal progenitors.

### mir-135a-2 overexpression in mir-135a-2^+/-^ mice does not result in forebrain abnormalities

**Fig. 7:**
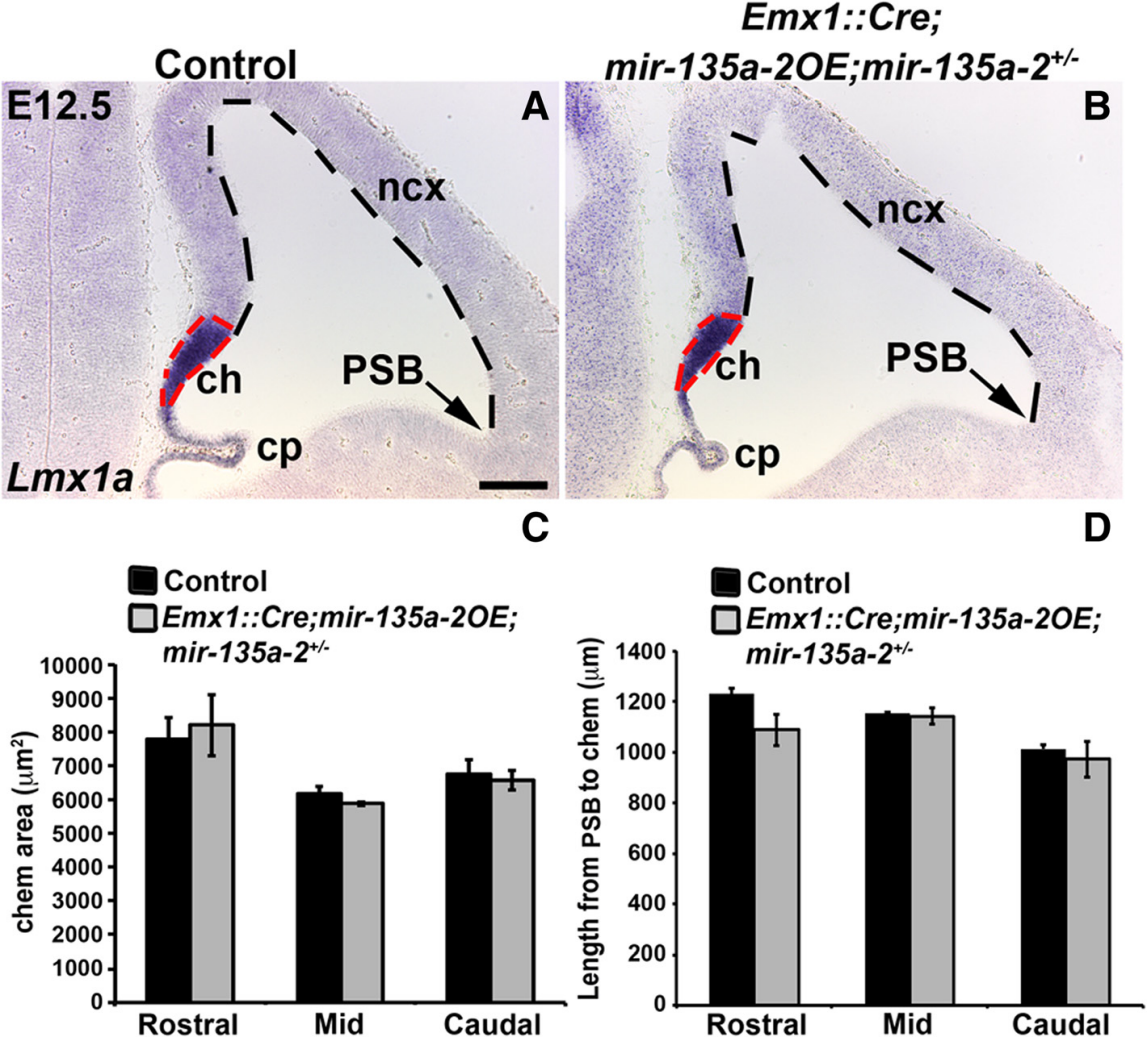
*mir-135a-2OE* in mice heterozygous for endogenous *mir-135a-2.* **a**-**b** *ish* showing *Lmx1a* expression in E12.5 control (*Emx1::Cre*;*mir-135a-2* ^+/-^) (**a**) and mutant (*Emx1::Cre*;*mir-135a-2OE*;*mir-135a-2* ^+/-^) (**b**) brains. Dashed lines highlight the cortical hem area and the neocortical domain size. **c** Quantification of the cortical hem area (μm^2^ ± SEM) along the rostro-caudal axis (*n* = 3). **d** Quantification of the neocortex length (μm ± SEM) from the PSB to the hem at the same levels (*n* = 3). ch, cortical hem; cp, choroid plexus; PSB, pallium-subpallium boundary; ncx, neocortex. Scale bar 100 μm in panels **a** and **b**

To further demonstrate the specificity of our results, we removed one copy of endogenous *mir-135a-2* from embryos conditionally overexpressing *mir-135a-2.* We reasoned that if the phenotype observed in *Emx1::Cre*;*mir-135a-2OE* mutants is due to *mir-135a-2* overexpression, then, removal of one copy of the endogenous *mir-135a-2* should alleviate this phenotype. E12.5 embryos of *Emx1::Cre*;*mir-135a-2OE*;*mir-135a-2*^+/-^ (mutants) and *Emx1::Cre*;*mir-135a-2*^+/-^ (controls) genotypes were analyzed for the expression of *Lmx1a* (Fig. 7a and b) and *Wnt3a* (Additional file 10: Figure S10). The cortical hem area and the extent of the cortical domain were quantified as previously described (Fig. 7c and d). No alterations in these domains and in the choroid plexus were observed, suggesting that normal *miR-135a* levels are important for embryonic forebrain development.

## Discussion

**Fig. 8:**
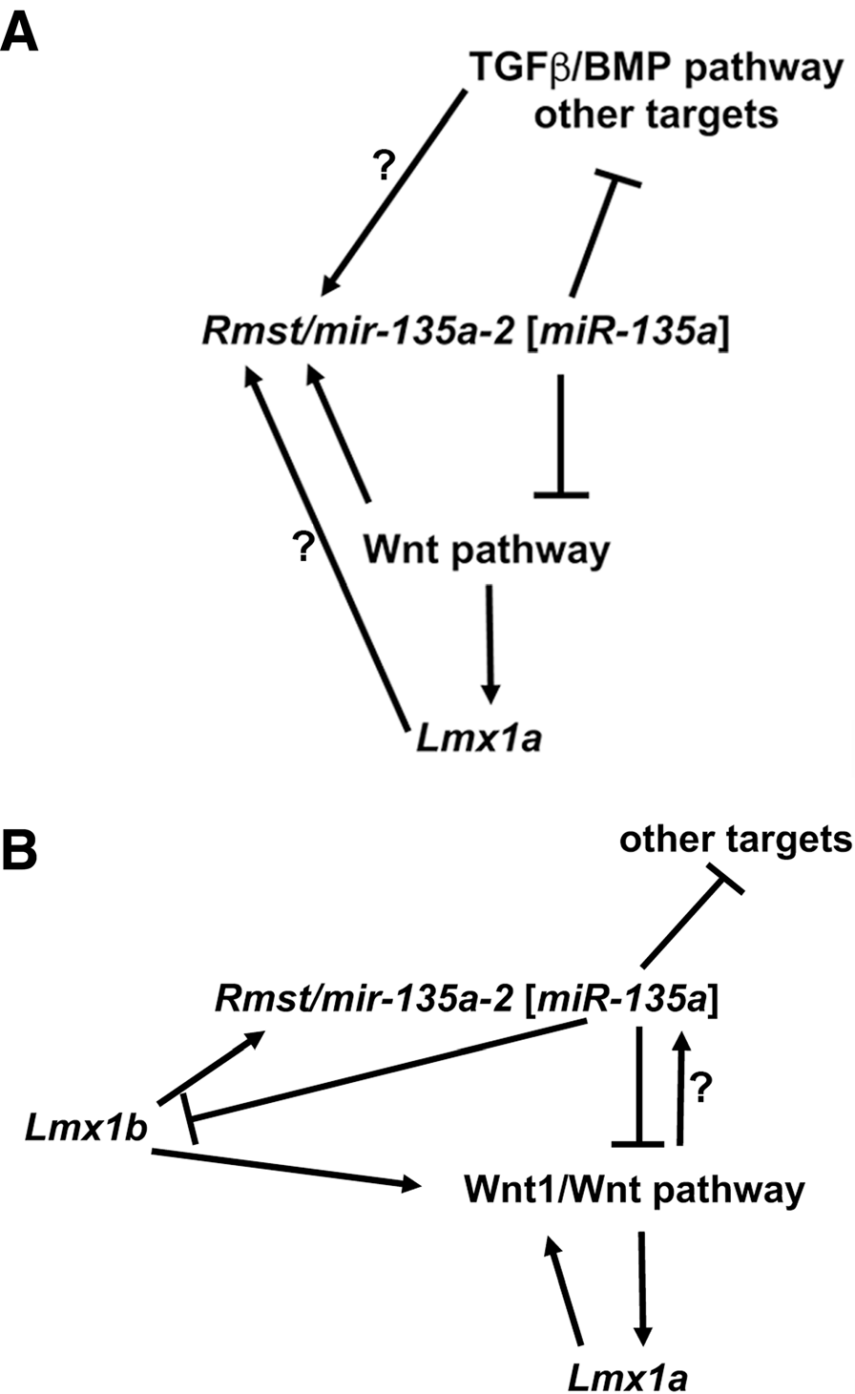
Schematic representation of interactions between *Lmx1* genes, the Wnt pathway and *Rmst/mir-135a-2 [miR-135a].* **a** In the embryonic forebrain, canonical Wnt/beta-catenin pathway induces *Rmst/mir-135a-2 [miR-135a]*, as well as the cortical hem determinant *Lmx1a. miR-135a*, in turn, negatively targets Wnt pathway mRNAs establishing an auto-regulatory loop. *miR-135a* is also predicted to target TGFβ/BMP pathway mRNAs as well as other targets. It is possible that the TGFβ/BMP pathway is also able to induce *Rmst/mir-135a-2 [miR-135a]* and establish a *miR-135a/*TGFβ/BMP auto-regulatory loop. It is also possible that *Lmx1a* contributes to *Rmst/mir-135a-2 [miR-135a]* expression. However, these interactions remain to be demonstrated. Ultimately, this complex network of positive and negative interactions plays a role in determining proper dorsal forebrain size (cortical hem, hippocampus primordium, choroid plexus and neocortex). **b** A similar scenario was previously identified in the embryonic midbrain [11] where *Lmx1b* drives the expression of Wnt1/Wnt pathway and of *Rmst/mir-135a-2 [miR-135a]*, which in turn negatively modulates the levels of *Lmx1b*, Wnt1/Wnt pathway and other targets. Additionally, Wnt1/Wnt pathway and *Lmx1a* interactions have been demonstrated. All together these interactions are critical for midbrain dopamine progenitor pool patterning and expansion

From the present study we deduced six prominent conclusions. First, *miR-135a,* and *Rmst*, are expressed and functional in the embryonic dorsal forebrain. Second, in the dorsal forebrain, mature *miR-135a* is predominantly derived from the *mir-135a-2* locus. Third, *miR-135a* is dispensable for forebrain development. Fourth, modest *mir-135a-2* overexpression, within an early but not late time window, results in a phenotype consistent with Wnt and TGFβ/BMP signaling deficits – a reduced cortical hem, hippocampus primordium and neocortex, and a less convoluted choroid plexus. Fifth, Wnt/beta-catenin pathway is necessary and sufficient to induce *Rmst* and *miR-135a* expression pointing to the existence of a *Wnt/miR-135a* auto-regulatory loop, which could serve to limit the extent, the duration and/or intensity of the Wnt pathway. Finally, Wnt/beta-catenin pathway is also necessary for *Lmx1a* expression, a key cortical hem determinant. A model of these interactions is depicted in Fig. 8.

Wnt and TGFβ/BMP morphogens act through gradients of signaling cascades and have been implicated in various facets of dorsal neural tube development [16, 19–22]. Several studies have suggested that the dosage of the Wnt and TGFβ/BMP pathways is critical and has to be tightly controlled through intricate networks of positive and negative feedback loops [15, 19, 23, 32, 33]. It is therefore challenging to understand how these pathways are modulated in time and space during embryonic development, how cells receive and integrate multiple signals and whether potential nodes of intersection exist. Recently, it has been demonstrated that microRNAs contribute to gene networks that transform the graded activity of a morphogen in robust cell fate decisions by establishing context-dependency, threshold responses and sharpening temporal and spatial expression patterns [4, 12, 72]. *miR-135a* and its host long non-coding transcript, *Rmst,* were shown to be expressed and functional in the embryonic Wnt-rich domain of the midbrain [11]. Here, we have shown that *miR-135a* and *Rmst* are also co-expressed in the Wnt and TGFβ/BMP-rich domains of the embryonic dorsal forebrain suggesting a correlation between this microRNA, the Wnts and TGFβ/BMPs-rich domains across the embryonic CNS. Interestingly, akin to several Wnts and TGFβ/BMPs, strong expression of *Rmst* in the embryonic hippocampal primordium declines over time and, at post-natal stages, become restricted to the fimbria and virtually undetectable in the adult hippocampus. *Rmst* expression has also been detected in human fetal cortical radial glia, suggesting a conserved role for this locus [73]. Taking together, the *Rmst/miR-135a* expression pattern in mice and humans, the finding that Wnt signaling is indeed able to induce their expression and the strong bioinformatics predictions, we postulate that this microRNA might play a role in fine-tuning the Wnt pathway and, possibly, the TGFβ/BMP pathway.

Embryos lacking *Wnt3a* [16]*,* functional LEF1 [66], or with genetic ablation of the cortical hem [44, 65], display loss of the hippocampus and shrinkage of the neocortex [44, 65], while mice with disrupted TGFβ/BMP signaling fail to develop or properly differentiate the choroid plexus [29]. Modest *mir-135a-2* overexpression appears to recapitulate these phenotypes, albeit in a milder fashion. Coupled with the findings that this microRNA is bioinformatically predicted to target both positive and negative regulators in the Wnt and TGFβ/BMP pathways, the Wnt related phenotypes observed in the midbrain [11], and proven interactions between *miR-135a* and targets like *GSK*, *Tcf7l2*, *Ccnd1* and *APC* in heterologous systems or cancer cell lines [11, 74–78], we posit that *miR-135a* modulates the Wnt and TGFβ/BMP signaling cascade in the developing forebrain.

*mir-135a-2* loss of function embryos did not display overt forebrain phenotypes, at least by the criteria that we assayed. One plausible explanation comes from the observation that members of a microRNA family are often predicted to target the same or overlapping sets of genes and therefore to act in a functionally redundant manner [63]. Supporting this idea, single microRNA loss of function or single microRNA silencing, through antisense oligonucleotides and sponge techniques, typically results in subtle or no phenotypes [64, 79–81]. Because *mir-135b* was expressed in the embryonic ventral forebrain, we ruled out any compensatory effect due to this microRNA. We considered the possibility that the other member of this family, *mir-135a-1* might be expressed in a similar and overlapping pattern. Because *mir-135a-1* and *mir-135a-2* produce an identical mature form, it is not possible to analyze their differential expression by standard techniques. However, RT-qPCR experiments for mature *miR-135a* on dorsal forebrain tissue of *mir-135a-2* null embryos revealed greatly reduced expression levels. Therefore, we concluded that the main *miR-135a* mature form in the dorsal forebrain could be attributed to *mir-135a-2* precursor. Redundancy, on the other hand, can either be attributed to very low levels of *miR-135a* produced by the *mir-135a-1* precursor or to some other microRNA having a similar seed sequence. This latter, more likely, possibility has been raised in the recent literature [82].

Identifying regulators of the Wnt pathway has been highlighted as an important goal [83]. *miR-135a* is up regulated in several tumor types characterized by high levels of Wnt/beta-catenin signaling, including colorectal tumors as well as certain subtypes of medulloblastomas [74, 76]. It is possible that, in these tumors, Wnt signaling, in accordance with our data in the forebrain, induces *miR-135a*. If so, *miR-135a* would serve as a useful marker for aberrant Wnt signaling in these tumors, and possibly others, and in therapeutic protocols designed to circumscribe unrestrained Wnt signaling. In colorectal cancers, *miR-135a* has been proposed to act as a positive regulator of Wnt signaling by targeting APC, a key molecule of the beta-catenin destruction complex [74]. Thus, while it is emerging that this microRNA is intimately correlated with Wnt signaling in the embryonic CNS ([11]and this work), in tumors [74–76] and in other cell types with high Wnt signaling [77, 78], it is possible that the net effect of *miR-135a* depends on the physiological milieu. Elucidating the targets may reveal how a single microRNA, predicted to target both positive and negative regulators of a signaling pathway, may have distinct outcomes depending on the context. Ultimately, an array of biochemical and genetic approaches will be required to accurately define direct *miR-135a* targets and the molecular underpinnings of *mir-135a-2* mutant phenotypes.

Finally, our study has highlighted a role for Wnt/beta-catenin signaling in the expression of cortical hem determinant *Lmx1a,* a LIM-homeodomain transcription factor.

Wnt1/Wnt signaling has been shown to form an auto-regulatory loop with *Lmx1a* to control dopaminergic neurons differentiation in the embryonic ventral midbrain [22, 23, 84, 85]. In the dorsal embryonic forebrain, *Lmx1a* is expressed in the cortical hem, a Wnt-rich region. While *Lmx1a* is not required for cortical hem induction, it is critical for proper regulation of cell fate decisions [58]. In the absence of *Lmx1a*, the hippocampal selector gene *Lhx2* is ectopically expressed in the cortical hem leading to excessive production of hippocampal cells and decreased production of Cajal-Retzius cells [58]. Here, through gain and loss of function experiments of beta-catenin, we show that, in the dorsal forebrain akin to the ventral midbrain [22, 24], Wnt/beta-catenin signaling is necessary, but not sufficient, for *Lmx1a* expression. Wnt/beta-catenin induction of *Lmx1a* is likely to be a key event in the proper cortical hem cell fate establishment and/or maintenance.

## Additional files

Additional file 1: Figure S1.
*Rmst* expression during forebrain development. (A-F) *Rmst ish* on coronal sections of wild type brains from E11.5 to adult stage. (A) At E11.5, *Rmst* is expressed in the epithalamus, medial wall of the dorsal telencephalon, the eminentia thalami, and the septum. (B) At E12.5 (as in Fig. 1), *Rmst* was detected in the medial wall of the telencephalon and in the diandephalon. (C) At E14.5, *Rmst* is still highly expressed in the cortical hem and hippocampus primordium, thalamus and hypothalamus. (D) At E16.5, *Rmst* expression is high in the hippocampus and fimbria but low in scattered cells of the migratory stream. Expression in the thalamus is maintained. (E) At post-natal stages P1, *Rmst* is restricted to the fimbria, yet expressed outside of the hippocampal formation to become virtually undetectable in the hippocampus of adult mice (F) (hippocampal staining is not considered specific to the *Rmst* probe). dm, dorso-medial wall of telencephalon; emT, eminentia thalami; Et, epithalamus; tel, telencephalon; v3, third ventricle; lv, lateral ventricle; Sp, septum; d, diencephalon; hp, hippocampus; ch, cortical hem; cp, choroid plexus; ncx, neocortex; LGE, lateral ganglionic eminences; th, thalamus, hy, hypothalamus; fm, fimbria; ms, migratory stream; CA1 and CA3, hippocampal fieds; DG, dentate gyrus. Scale bar 400 μm in panels A, C and D; 100 μm in panel B; 200 μm in panels E and F. (TIF 1931 kb) Additional file 2: Figure S2.
*Wnt3a* expression in mice with elevated beta-catenin. (A-B) *Wnt3a in situ *hybridization on control (A) and mutant (*Emx1::Cre;Ctnnb1* ^*lox(ex3*)^) E12.5 coronal sections. Brackets highlight the cortical hem. ch, cortical hem; cp, choroid plexus. Scale bar 100 μm. (TIF 2822 kb) Additional file 3: Figure S3.
*miR-135a* bioinformatics analysis. A) Top pathways targeted by *miR-135a*. TGFβ/BMP and Wnt signaling pathways rank at position number 2 and 3 with high statistical significance. P values and number of genes targeted in each pathway are indicated. B) Overview of TGFβ/BMP cascade with highlighted, and listed, 16 genes targeted by *miR-135a*. It is worth noticing that the number of listed genes reflects only the data available on the Diana web site and the algorithm used for the search, as a higher number of putative *miR-135a* targets have already been reported using multiple search engines [11]. (TIF 1865 kb) Additional file 4: Figure S4.
*miR-135a* targets several mRNAs in the Wnt pathway. Schematic representation of *miR-135a* predicted targets in the Wnt signaling pathway, as generated by the Diana-miRPath software [52]. The 22 genes predicted to be *miR-135a* targets are highlighted and listed. As for the TGFβ/BMP pathway, the number of genes here listed reflects the data available on the Diana web site and the algorithm used for the search. (TIF 1741 kb) Additional file 5: Figure S5. Quantification of *miR-135a* expression in *Emx1::Cre;mir-135a-2OE* mice. RT-qPCR showing a 1.5 fold change in *miR-135a* expression level in E12.5 dissected dorsal forebrain tissue from mutant mice compared to controls (*n* = 3). Data are shown as a fold change and have been normalized to microRNA *sno202*. ***, *p* value <0.001. (TIF 145 kb) Additional file 6: Figure S6. Early *mir-135a-2* overexpression affects choroid plexus development. (A-B) Coronal sections of E12.5 control (*mir-135a2-OE*) and mutant brains (*Emx1*::Cre;*mir-135a-2OE*) showing expression of choroid plexus specific marker *rTtr1*. The dashed red line is used to highlight the change in choroid plexus complexity. cp, choroid plexus. Scale bar 100 μm. (TIF 2429 kb) Additional file 7: Figure S7. Early *mir-135a-2* overexpression affects hippocampus development. (A-D) Coronal sections of post-natal stage P1 and adult brains processed for *ish* for *NeuroD2*. Panels A, B show overall morphology and size of the hippocampus. Asterisks point towards the pallium-subpallium boundary. Insets show the extent of the hippocampus from the CA1 field to the tip of the dentate gyrus, quantification of which, at mid and caudal level of the brain, is reported in panel E. Length is expressed in μm ± SEM (*n* = 4). Consistent with a reduced cortical hem size at embryonic stages, the hippocampus was significantly reduced in its extent in mutant brains with respect to controls. ***, *p* <0.001. Abbreviations: pSub, para subiculum; Sub, subiculum; DG, dentate gyrus; Cng, cingulate cortex. Scale bar 400 μm in panels A and B; 200 μm in panels C and D. (TIF 2162 kb) Additional file 8: Figure S8. Expression of bioinformatically predicted *miR-135a* targets. (A-D) Coronal sections of E12.5 controls (*mir-135a-2OE*) and mutant (*Emx1::Cre;mir-135a2-OE*) brains, processed for immunohistochemistry (IHC) (A-B) or in situ hybridization (C, D). phospho-Smad (1/5/8) IHC on control (A) and mutant (B) brain coronal sections shows reduced signal in the cortical hem and the choroid plexus domains of mutants (arrowheads). Panels C and D show a reduced *Msx2* expression domain. ch, cortical hem; cp, choroid plexus. Scale bar 25 μm in panels A and B; 50 μm in panels C and D. (TIF 5061 kb) Additional file 9: Figure S9. Late *mir-135a-2* overexpression does not overtly affect forebrain development. Coronal sections of E13.5 control (*mir-135a-2*OE) and mutant brains (*Nes::Cre;mir-135a-2OE*) processed for *in situ* hybridization for *Lmx1a*. Little to no change was observed in the cortical hem and neocortical domain (dashed lines) or in the choroid plexus. ch, cortical hem; cp, choroid plexus; ncx, neocortex. Scale bar 400 μm. (TIF 2507 kb) Additional file 10: Figure S10. Little to no change in cortical hem of *Emx1::Cre*;*mir-135a-2OE*;*mir-135a-2* ^+/-^ mutant mice. (A-B) *ish* showing cortical hem marker *Wnt3a* in E12.5 control (*Emx1::Cre*;*mir-135a-2* ^+/-^) (A) and mutant (*Emx1::Cre*;*mir-135a-2*OE;*mir-135a-2* ^+/-^) (B) brains. ch, cortical hem. Scale bar 100 μm. (TIF 542 kb)
