# Supplementary figures and images for: Expression and functional analysis of the Wnt/beta-catenin induced mir-135a-2 locus in embryonic forebrain development
Source: Neural Dev. 2016 Apr 5;11:9. doi: 10.1186/s13064-016-0065-y (PMC4822265; doi:10.1186/s13064-016-0065-y)

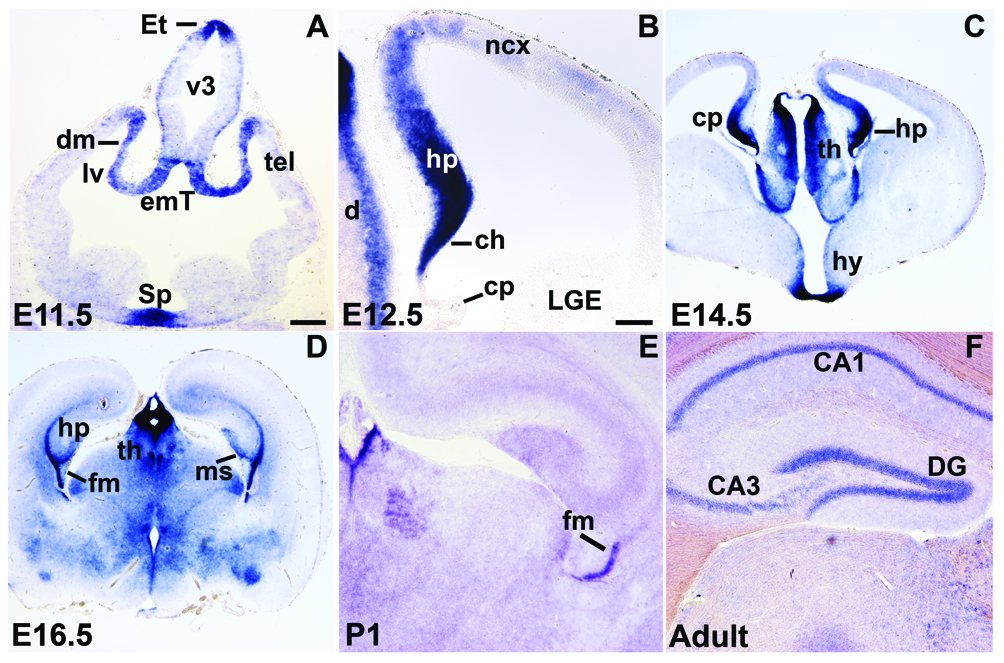

Supplement: Additional file 1: Figure S1. — Rmst expression during forebrain development. (A-F) Rmst ish on coronal sections of wild type brains from E11.5 to adult stage. (A) At E11.5, Rmst is expressed in the epithalamus, medial wall of the dorsal telencephalon, the eminentia thalami, and the septum. (B) At E12.5 (as in Fig. 1), Rmst was detected in the medial wall of the telencephalon and in the diandephalon. (C) At E14.5, Rmst is still highly expressed in the cortical hem and hippocampus primordium, thalamus and hypothalamus. (D) At E16.5, Rmst expression is high in the hippocampus and fimbria but low in scattered cells of the migratory stream. Expression in the thalamus is maintained. (E) At post-natal stages P1, Rmst is restricted to the fimbria, yet expressed outside of the hippocampal formation to become virtually undetectable in the hippocampus of adult mice (F) (hippocampal staining is not considered specific to the Rmst probe). dm, dorso-medial wall of telencephalon; emT, eminentia thalami; Et, epithalamus; tel, telencephalon; v3, third ventricle; lv, lateral ventricle; Sp, septum; d, diencephalon; hp, hippocampus; ch, cortical hem; cp, choroid plexus; ncx, neocortex; LGE, lateral ganglionic eminences; th, thalamus, hy, hypothalamus; fm, fimbria; ms, migratory stream; CA1 and CA3, hippocampal fieds; DG, dentate gyrus. Scale bar 400 μm in panels A, C and D; 100 μm in panel B; 200 μm in panels E and F. (TIF 1931 kb) [file 13064_2016_65_MOESM1_ESM.tif]

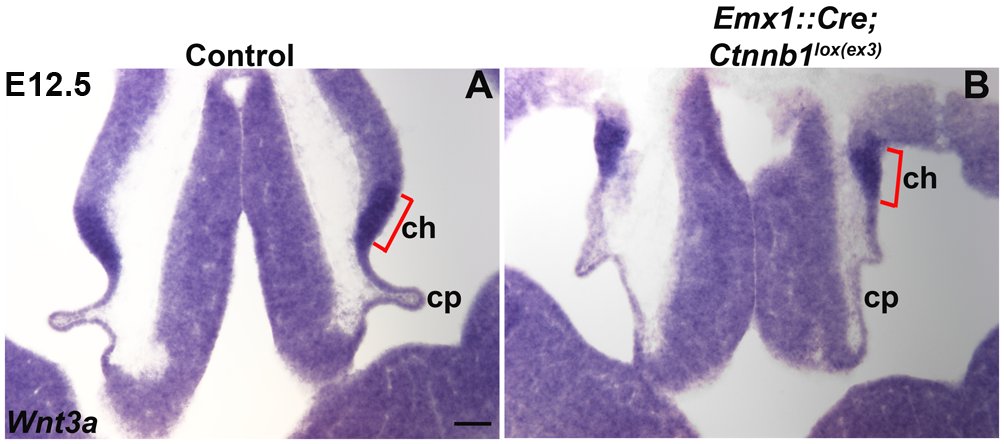

Supplement: Additional file 2: Figure S2. — Wnt3a expression in mice with elevated beta-catenin. (A-B) Wnt3a in situ hybridization on control (A) and mutant (Emx1::Cre;Ctnnb1 lox(ex3)) E12.5 coronal sections. Brackets highlight the cortical hem. ch, cortical hem; cp, choroid plexus. Scale bar 100 μm. (TIF 2822 kb) [file 13064_2016_65_MOESM2_ESM.tif]

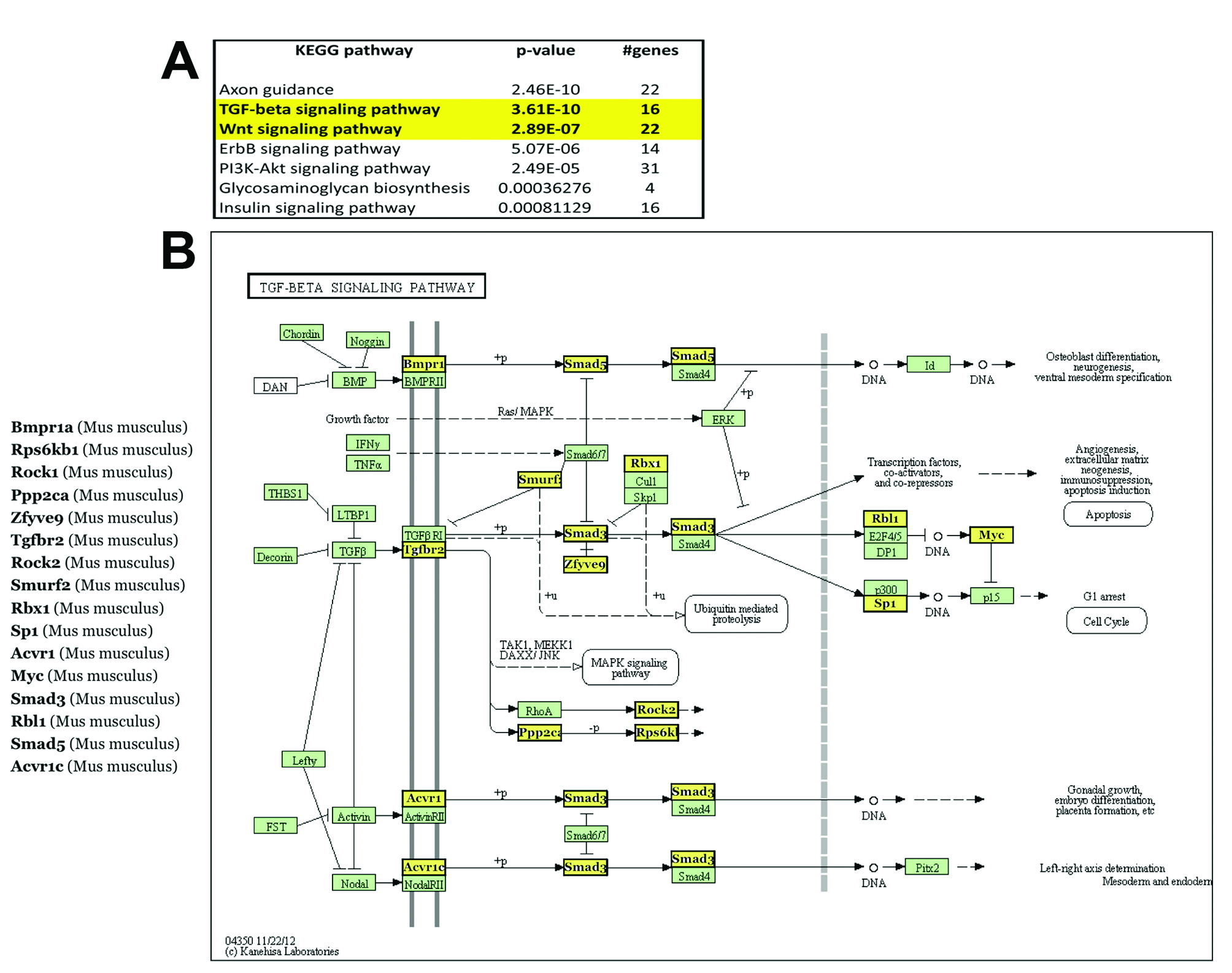

Supplement: Additional file 3: Figure S3. — miR-135a bioinformatics analysis. A) Top pathways targeted by miR-135a. TGFβ/BMP and Wnt signaling pathways rank at position number 2 and 3 with high statistical significance. P values and number of genes targeted in each pathway are indicated. B) Overview of TGFβ/BMP cascade with highlighted, and listed, 16 genes targeted by miR-135a. It is worth noticing that the number of listed genes reflects only the data available on the Diana web site and the algorithm used for the search, as a higher number of putative miR-135a targets have already been reported using multiple search engines [11]. (TIF 1865 kb) [file 13064_2016_65_MOESM3_ESM.tif]

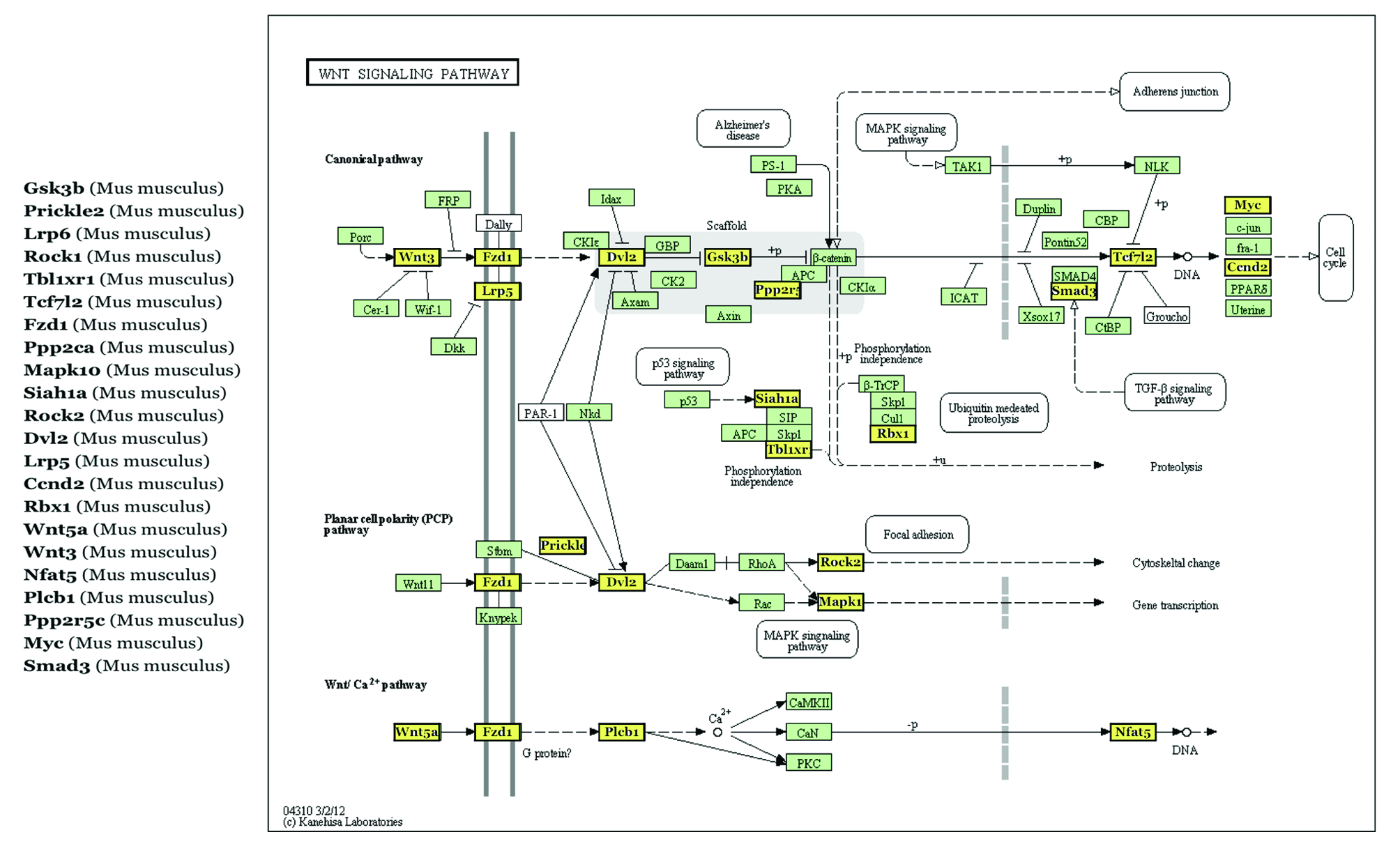

Supplement: Additional file 4: Figure S4. — miR-135a targets several mRNAs in the Wnt pathway. Schematic representation of miR-135a predicted targets in the Wnt signaling pathway, as generated by the Diana-miRPath software [52]. The 22 genes predicted to be miR-135a targets are highlighted and listed. As for the TGFβ/BMP pathway, the number of genes here listed reflects the data available on the Diana web site and the algorithm used for the search. (TIF 1741 kb) [file 13064_2016_65_MOESM4_ESM.tif]

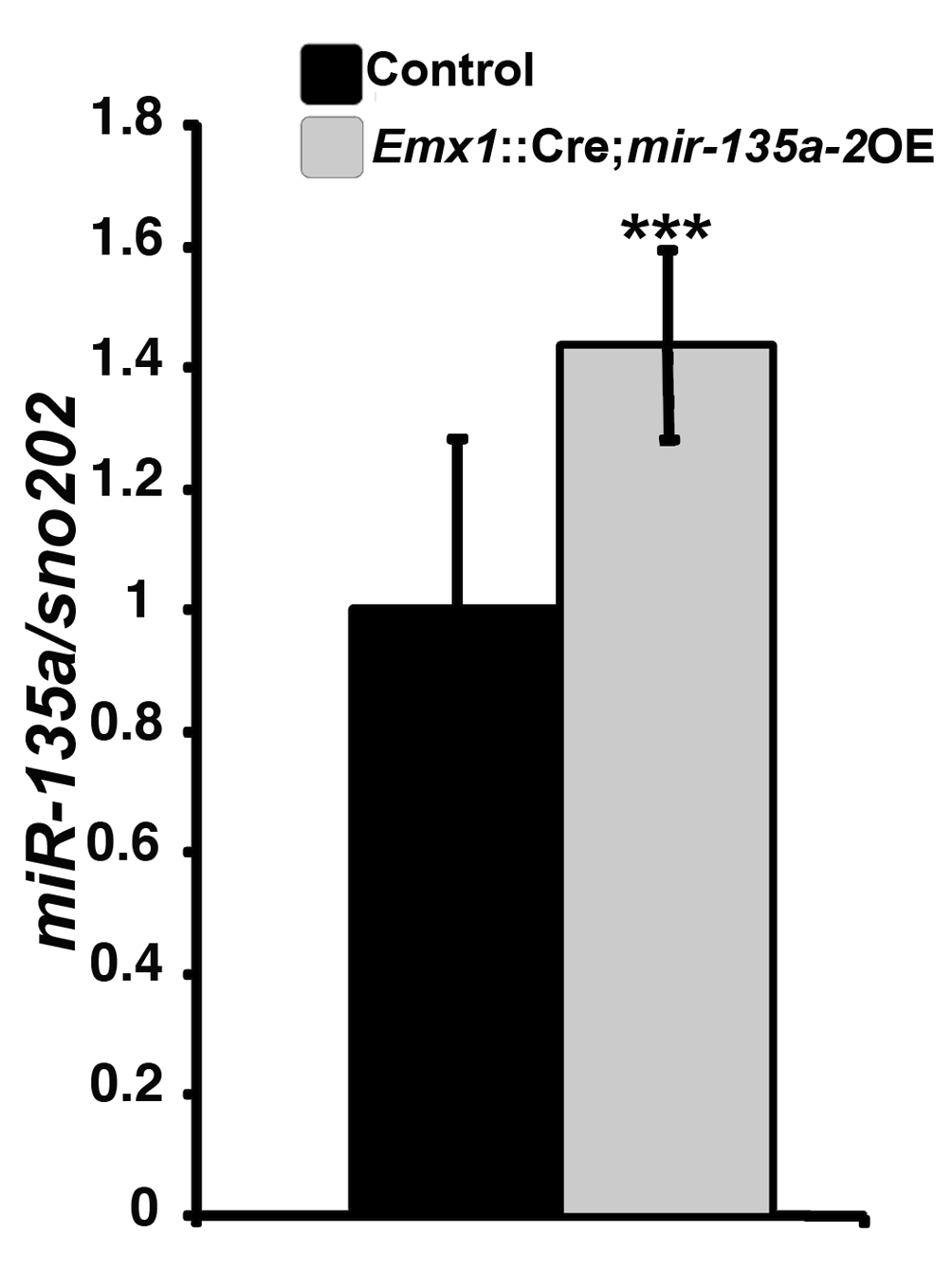

Supplement: Additional file 5: Figure S5. — Quantification of miR-135a expression in Emx1::Cre;mir-135a-2OE mice. RT-qPCR showing a 1.5 fold change in miR-135a expression level in E12.5 dissected dorsal forebrain tissue from mutant mice compared to controls (n = 3). Data are shown as a fold change and have been normalized to microRNA sno202. ***, p value <0.001. (TIF 145 kb) [file 13064_2016_65_MOESM5_ESM.tif]

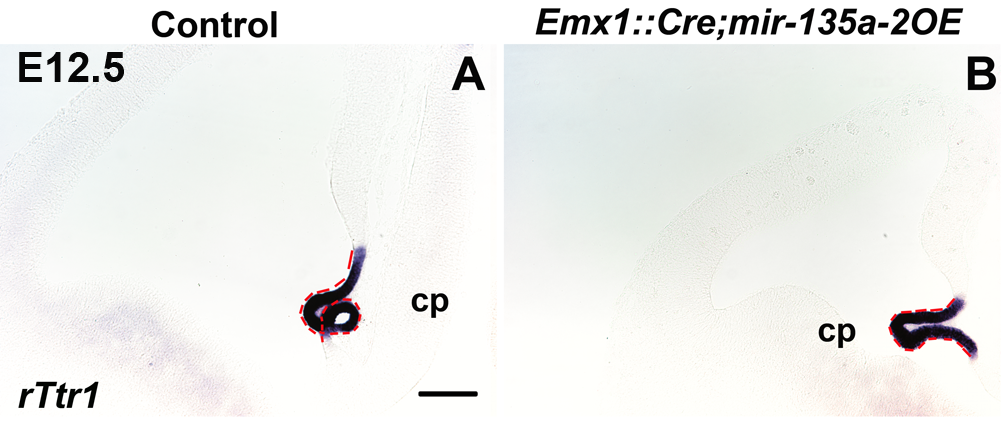

Supplement: Additional file 6: Figure S6. — Early mir-135a-2 overexpression affects choroid plexus development. (A-B) Coronal sections of E12.5 control (mir-135a2-OE) and mutant brains (Emx1::Cre;mir-135a-2OE) showing expression of choroid plexus specific marker rTtr1. The dashed red line is used to highlight the change in choroid plexus complexity. cp, choroid plexus. Scale bar 100 μm. (TIF 2429 kb) [file 13064_2016_65_MOESM6_ESM.tif]

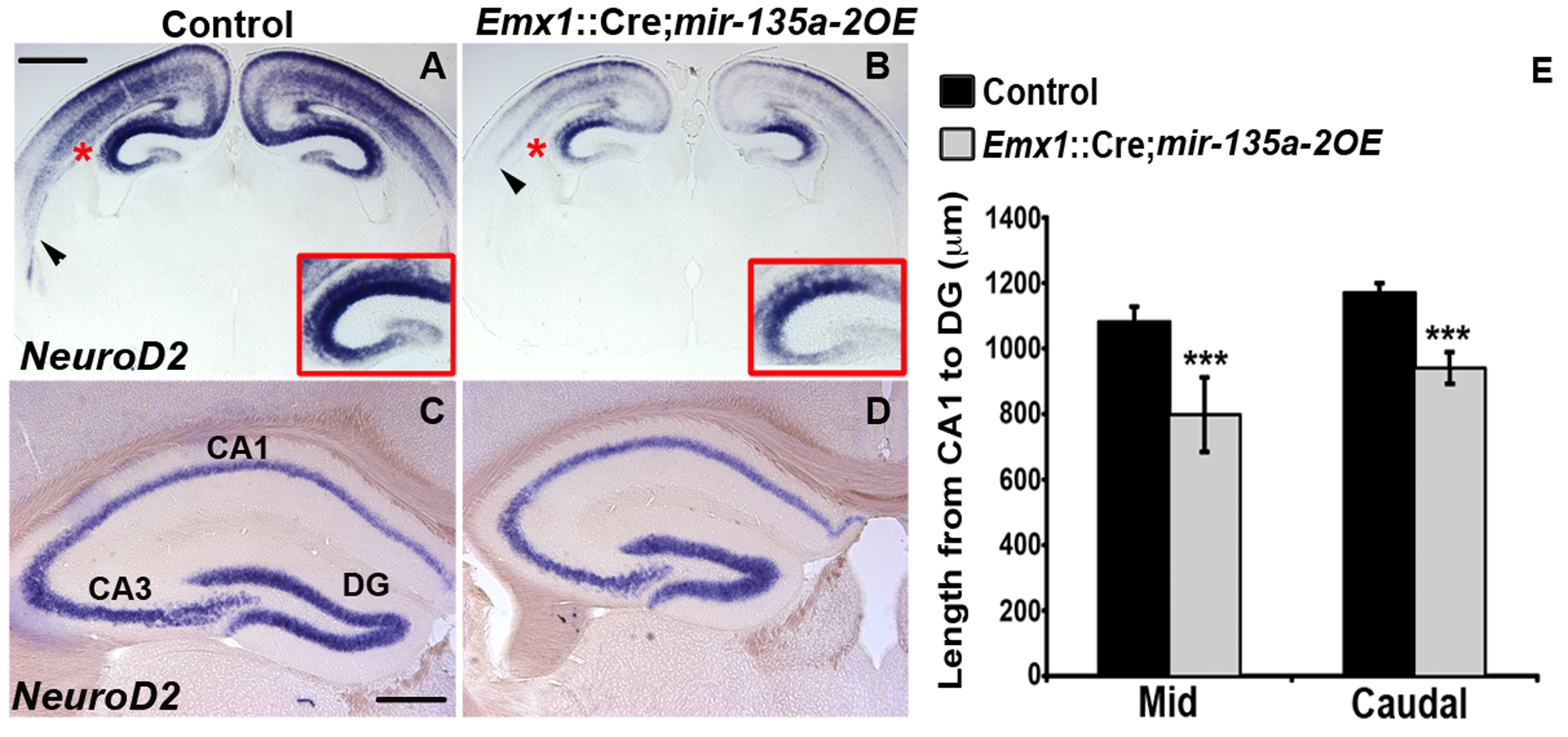

Supplement: Additional file 7: Figure S7. — Early mir-135a-2 overexpression affects hippocampus development. (A-D) Coronal sections of post-natal stage P1 and adult brains processed for ish for NeuroD2. Panels A, B show overall morphology and size of the hippocampus. Asterisks point towards the pallium-subpallium boundary. Insets show the extent of the hippocampus from the CA1 field to the tip of the dentate gyrus, quantification of which, at mid and caudal level of the brain, is reported in panel E. Length is expressed in μm ± SEM (n = 4). Consistent with a reduced cortical hem size at embryonic stages, the hippocampus was significantly reduced in its extent in mutant brains with respect to controls. ***, p <0.001. Abbreviations: pSub, para subiculum; Sub, subiculum; DG, dentate gyrus; Cng, cingulate cortex. Scale bar 400 μm in panels A and B; 200 μm in panels C and D. (TIF 2162 kb) [file 13064_2016_65_MOESM7_ESM.tif]

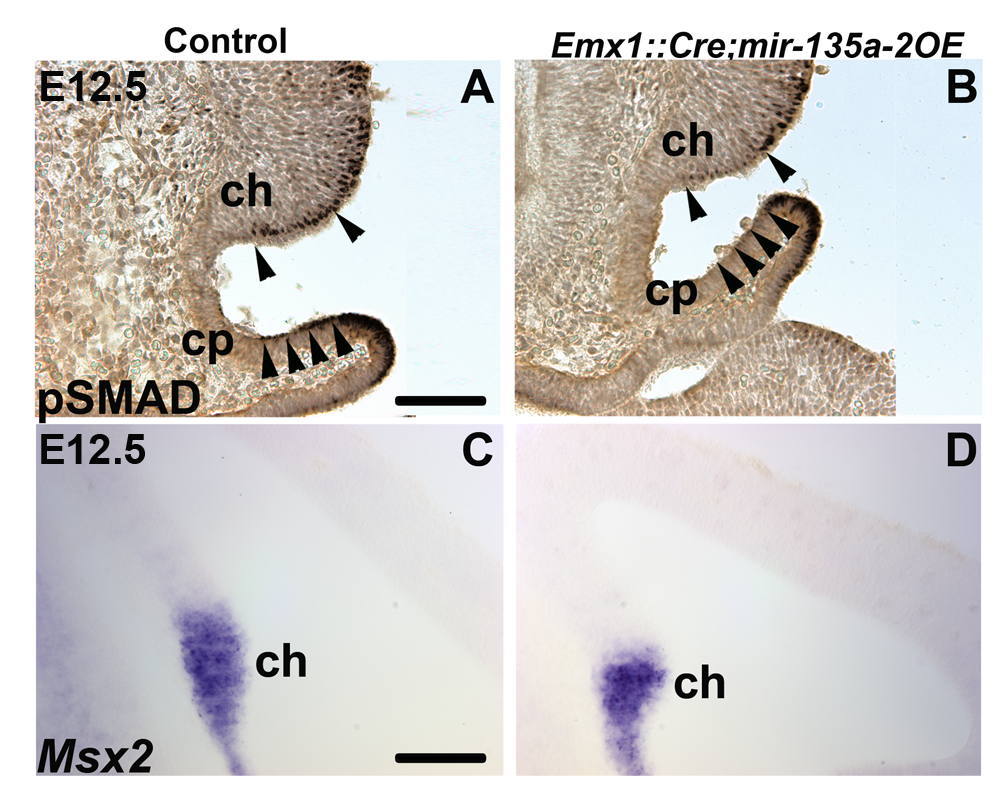

Supplement: Additional file 8: Figure S8. — Expression of bioinformatically predicted miR-135a targets. (A-D) Coronal sections of E12.5 controls (mir-135a-2OE) and mutant (Emx1::Cre;mir-135a2-OE) brains, processed for immunohistochemistry (IHC) (A-B) or in situ hybridization (C, D). phospho-Smad (1/5/8) IHC on control (A) and mutant (B) brain coronal sections shows reduced signal in the cortical hem and the choroid plexus domains of mutants (arrowheads). Panels C and D show a reduced Msx2 expression domain. ch, cortical hem; cp, choroid plexus. Scale bar 25 μm in panels A and B; 50 μm in panels C and D. (TIF 5061 kb) [file 13064_2016_65_MOESM8_ESM.tif]

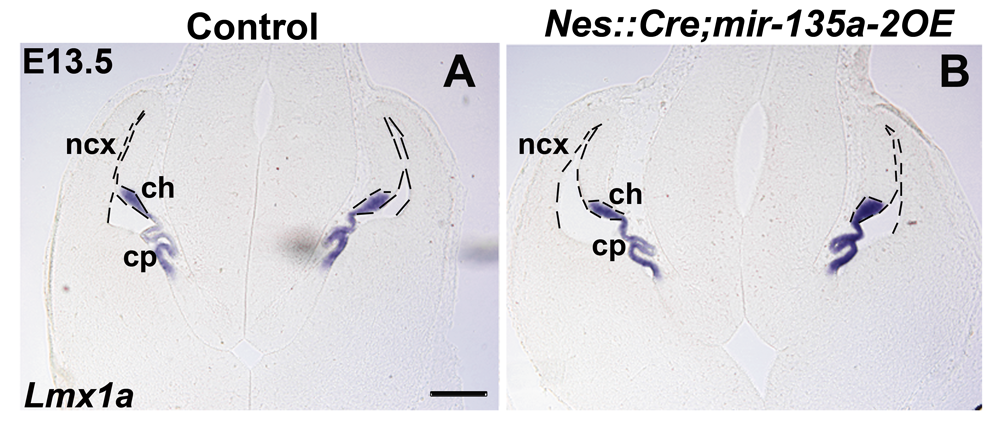

Supplement: Additional file 9: Figure S9. — Late mir-135a-2 overexpression does not overtly affect forebrain development. Coronal sections of E13.5 control (mir-135a-2OE) and mutant brains (Nes::Cre;mir-135a-2OE) processed for in situ hybridization for Lmx1a. Little to no change was observed in the cortical hem and neocortical domain (dashed lines) or in the choroid plexus. ch, cortical hem; cp, choroid plexus; ncx, neocortex. Scale bar 400 μm. (TIF 2507 kb) [file 13064_2016_65_MOESM9_ESM.tif]

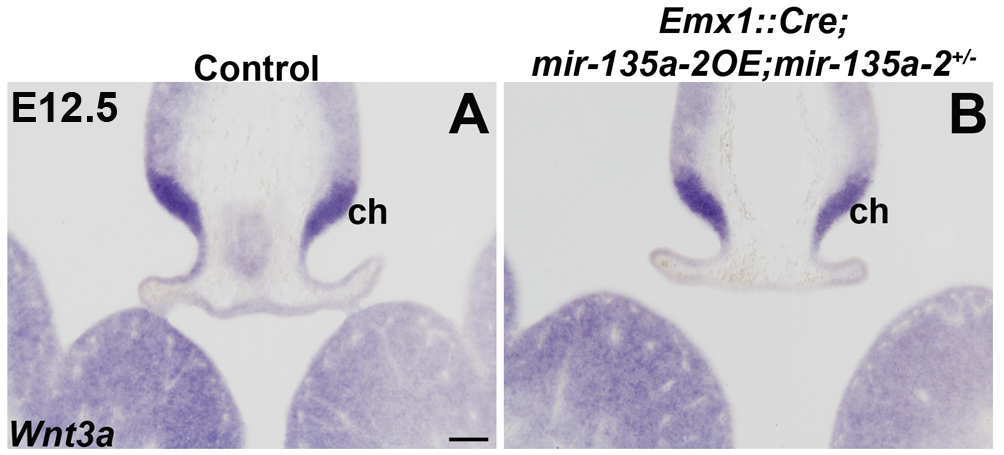

Supplement: Additional file 10: Figure S10. — Little to no change in cortical hem of Emx1::Cre;mir-135a-2OE;mir-135a-2 +/- mutant mice. (A-B) ish showing cortical hem marker Wnt3a in E12.5 control (Emx1::Cre;mir-135a-2 +/-) (A) and mutant (Emx1::Cre;mir-135a-2OE;mir-135a-2 +/-) (B) brains. ch, cortical hem. Scale bar 100 μm. (TIF 542 kb) [file 13064_2016_65_MOESM10_ESM.tif]
